# Supplementary material for: Bicarbonate signalling via G protein-coupled receptor regulates ischaemia-reperfusion injury
Source: Nat Commun. 2024 Feb 27;15:1530. doi: 10.1038/s41467-024-45579-3 (PMC10899177; doi:10.1038/s41467-024-45579-3)

Supplementary Information

Bicarbonate signalling via G protein-coupled receptor regulates ischaemia-reperfusion injury

Airi Jo-Watanabe[1, 2*], Toshiki Inaba[3], Takahiro Osada[4], Ryota Hashimoto[5], Tomohiro Nishizawa[6], Toshiaki Okuno[1], Sayoko Ihara[7], Kazushige Touhara[7], Nobutaka Hattori[3, 8], Masatsugu Oh-Hora[1, 5], Osamu Nureki[9], and Takehiko Yokomizo[1*]

Affiliations

[1]Department of Biochemistry, Juntendo University Graduate School of Medicine, Tokyo 113-8421, Japan

[2]AMED-PRIME, Japan Agency for Medical Research and Development, Tokyo 100-0004, Japan

[3]Department of Neurology, Juntendo University School of Medicine, Tokyo 113-8421, Japan

[4]Department of Neurophysiology, Juntendo University School of Medicine, Tokyo 113-8421, Japan

[5]Laboratory of Cell Biology, Biomedical Research Core Facilities, Juntendo University Graduate School of Medicine, Tokyo 113-8421, Japan

[6]Graduate School of Medical Life Science, Yokohama City University, Kanagawa 230-0045, Japan

[7]Department of Applied Biological Chemistry, Graduate School of Agricultural and Life Sciences, The University of Tokyo, Tokyo 113-8657, Japan

[8]Neurodegenerative Disorders Collaborative Laboratory, RIKEN Center for Brain Science, Saitama 351-0198, Japan

[9]Department of Biological Sciences, Graduate School of Science, The University of Tokyo, Tokyo 113-0033, Japan

***Corresponding Authors:**

Airi Jo-Watanabe, MD, PhD

2-1-1 Hongo, Bunkyo-Ku, Tokyo 113-8421, Japan

E-mail: awatanabe-tky@umin.ac.jp

Takehiko Yokomizo, MD, PhD

2-1-1 Hongo, Bunkyo-Ku, Tokyo 113-8421, Japan

E-mail: yokomizo-tky@umin.ac.jp

Supplementary Figures

Supplementary Fig. 1

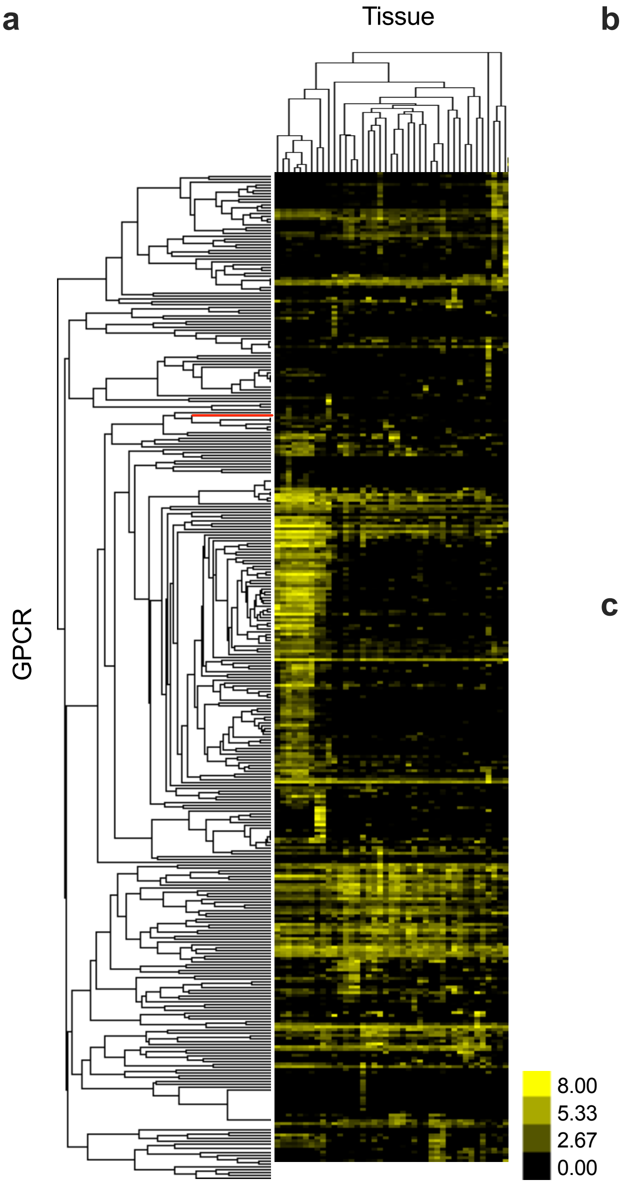

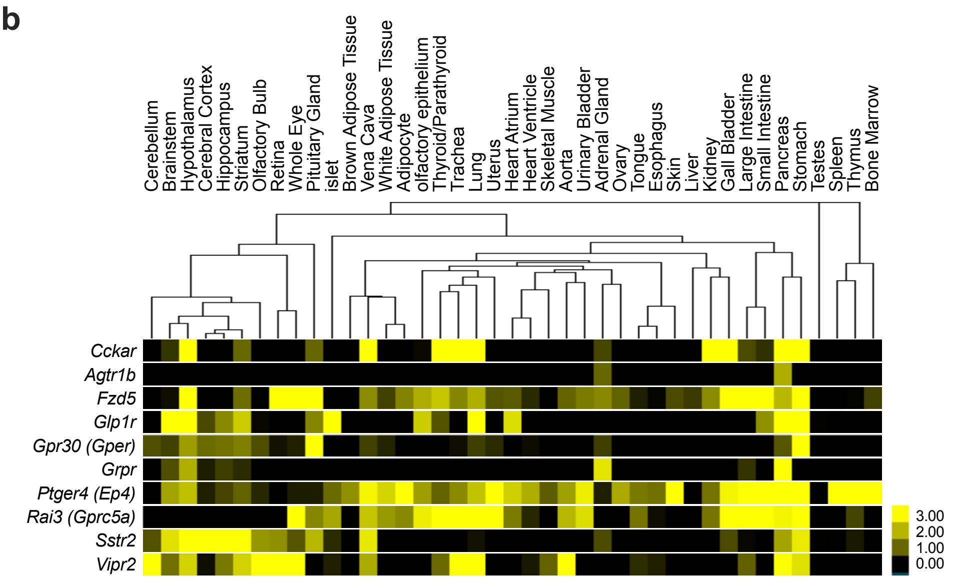

c

Gene	Ptorein	Ligand	Function	Gastroin testinal hormon	vasoacti vity	Wnt signal	high expression in the brain
Cckar	Cholecystokinin receptor type A	cholecystokinin	Mediates pancreatic growth and enzyme secretion, smooth muscle contraction of the gall bladder and stomach.	x			
Agtr1b	Type-1B angiotensin II receptor	angiotensin II	Mediates smooth muscle contraction of vasculature, cell growth, secretion of catecholamines and aldosterone.		x		x
Fzd5	Frizzled-5	Wnt proteins	Activates WNT2, WNT10B, WNT5A.Functions in the canonical Wnt/beta-catenin signaling pathway. Promotes formation of synapses in neurons.			x	
Glp1r	Glucagon-like peptide 1 receptor	glucagon-like peptide 1 (GLP-1)	Regulates insulin secretion in response to GLP-1.	x			
Gpr30 (Gper)	G-protein coupled estrogen receptor 1	17-β-estradiol (E_2)	Mediates pleiotropic functions in the cardiovascular, endocrine, reproductive, immune, and central nervous systems.		x		x
Grpr	Gastrin-releasing peptide receptor	gastrin-releasing peptide (GRP)	Regulates food intake, nonhistaminergic itch sensation	x			x
Ptger4 (Ep4)	Prostaglandin E2 receptor EP4 subtype	prostaglandin E2 (PGE2)	Relaxing effect on smooth muscle. Related to inflammation and cancer.		x		
Rai3 (Gprc5a)	Retinoic acid-induced protein 3	orphan	Interacts with frizzled GPCRs and activates the Wnt signaling pathway. A negative modulator of EGFR signaling.			x	
Sstr2	Somatostatin receptor type 2	somatostatin-14, -28	Functionally dominant isotype in pancreatic α– and β–cells. Inhibitory effect on hormone secretion. Stimulates neuronal migration and axon outgrowth.	x			x
Vipr2	Vasoactive intestinal polypeptide receptor 2	VIP, PACAP-38 and -27	Mediates smooth muscle relaxation, digestive enzyme secretion, and vasodilation. Regulates circadian rhythm.	x	x		

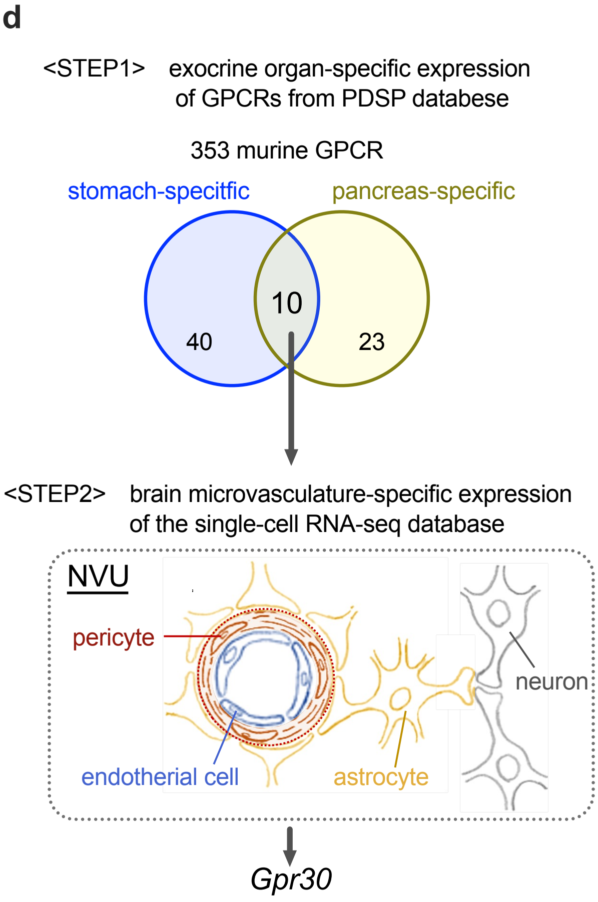

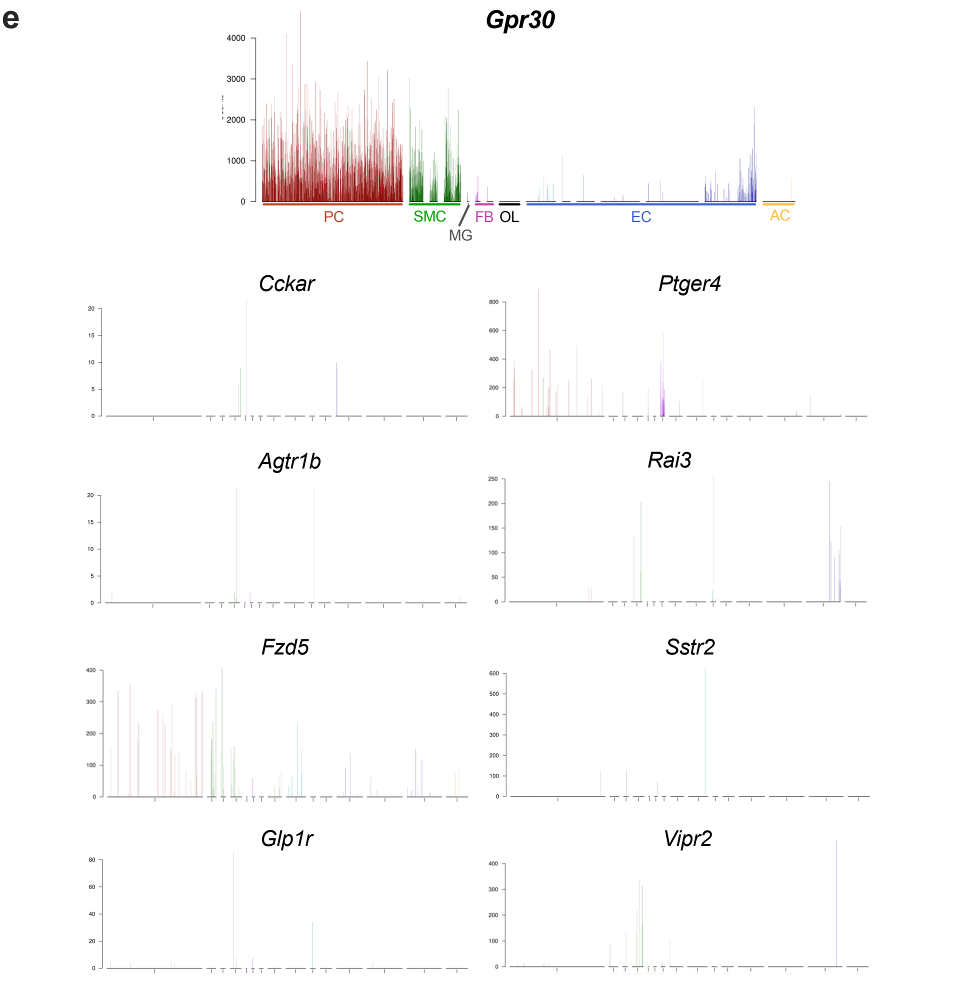

Supplementary Fig. 1. Expression profiles of various GPCRs and GPR30 in mouse tissues.

a) Public data from the PDSP database (https://pdsp.unc.edu/databases/ShaunCell/heatmaps.php) that covers the expression of 353 murine GPCRs in various tissues. The red line represents GPR30. **b)** Candidate GPCRs that are specifically expressed in the stomach and pancreas. **c)** Receptor names, ligands, and characteristics of the 10 candidate GPCRs. **'**High expression in the brain' indicates that the brain expresses a particular GPCR over the 10th highest level among other tissues. **d)** Two-step selection of candidate GPCRs. Step 1: Of the 353 murine GPCRs, 50 and 33 were highly expressed in stomach and pancreas, respectively. From these, 10 GPCRs that were commonly highly expressed in both the tissues were selected as potential candidates. Among the 10 candidate GPCRs, GPR30 was selected for the neurovascular unit (NVU) -specific expression in the brain. **e)** Expression of each indicated candidate GPCR in single-cell components of the NVU. PC, pericytes; SMC, smooth muscle cells; MG, microglia; FB, vascular fibroblast-like cells; OL, oligodendrocytes; EC, endothelial cells; AC, astrocytes. Data were obtained from the single-cell RNA-sequencing database of mouse brain vascular and perivascular cells (http://betsholtzlab.org/VascularSingleCells/database.html).

Supplementary Fig. 2

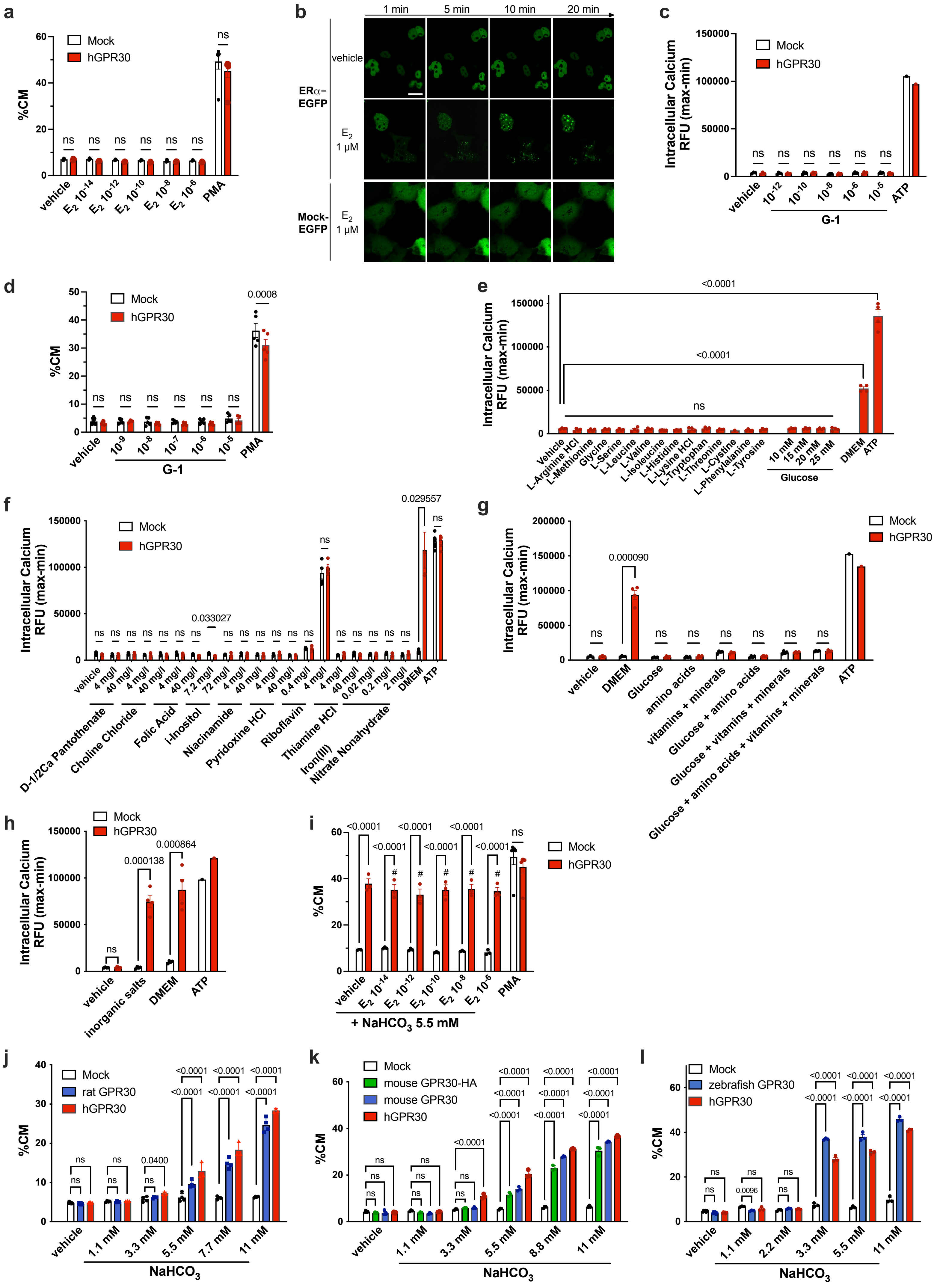

Supplementary Fig. 2. Activation of GPR30 by bicarbonate ions.

a) TGFα shedding assay using mock- and human GPR30 (hGPR30)-transfected HEK293 cells treated with oestradiol (E_2). PMA (100 nM) was used as the positive control. **b)** E_2-dependent accumulation of oestrogen receptor α (ERα). COS-7 cells expressing ERα fused with EGFP (ERα-EGFP) or mock-EGFP were treated with vehicle or E_2 (1 µM) and examined using confocal microscopy. Mock-EGFP served as the negative control. Scale bar, 20 µm. **c, d)** The calcium mobilization assay (c) and the TGFα shedding assay (d) using hGPR30-transfected HEK293 cells treated with a putative synthetic agonist, G-1. ATP (25 µM) and PMA (100 nM) were used as positive controls. **e–h)** Inorganic components of DMEM activate hGPR30. Amino acids and glucose (e), vitamins, and trace elements (f) in DMEM, and their combination (g) did not induce calcium mobilization in MCF-GPR30. A mixed solution of the inorganic components of Dulbecco's modified Eagle's medium (DMEM) elevated intracellular calcium (h). **i)** hGPR30-transfected HEK293 cells were treated with 5.5 mM sodium bicarbonate and the indicated concentrations of E_2 in the TGFα shedding assay. **j–l)** TGFα shedding assay using HEK293 cells transfected with rat (j), mouse (k), and zebrafish (l) GPR30, and treated with physiological concentrations of bicarbonate ions. Statistical analysis: two-tailed unpaired t-test with Bonferroni's correction after two-way ANOVA (**a, c, d, i–l**). In **i**, # indicates no significant difference compared with the hGPR30 column treated with the vehicle. Two-tailed unpaired t-test with Bonferroni's correction after one-way ANOVA **(e)**. Two-tailed unpaired t-test with Holm-Šídák's correction **(f–h)**. Data are presented as mean values ± SEM. *P* values are shown if significant. ns indicates no significant difference. Source data are provided as a Source Data file.

Supplementary Fig. 3

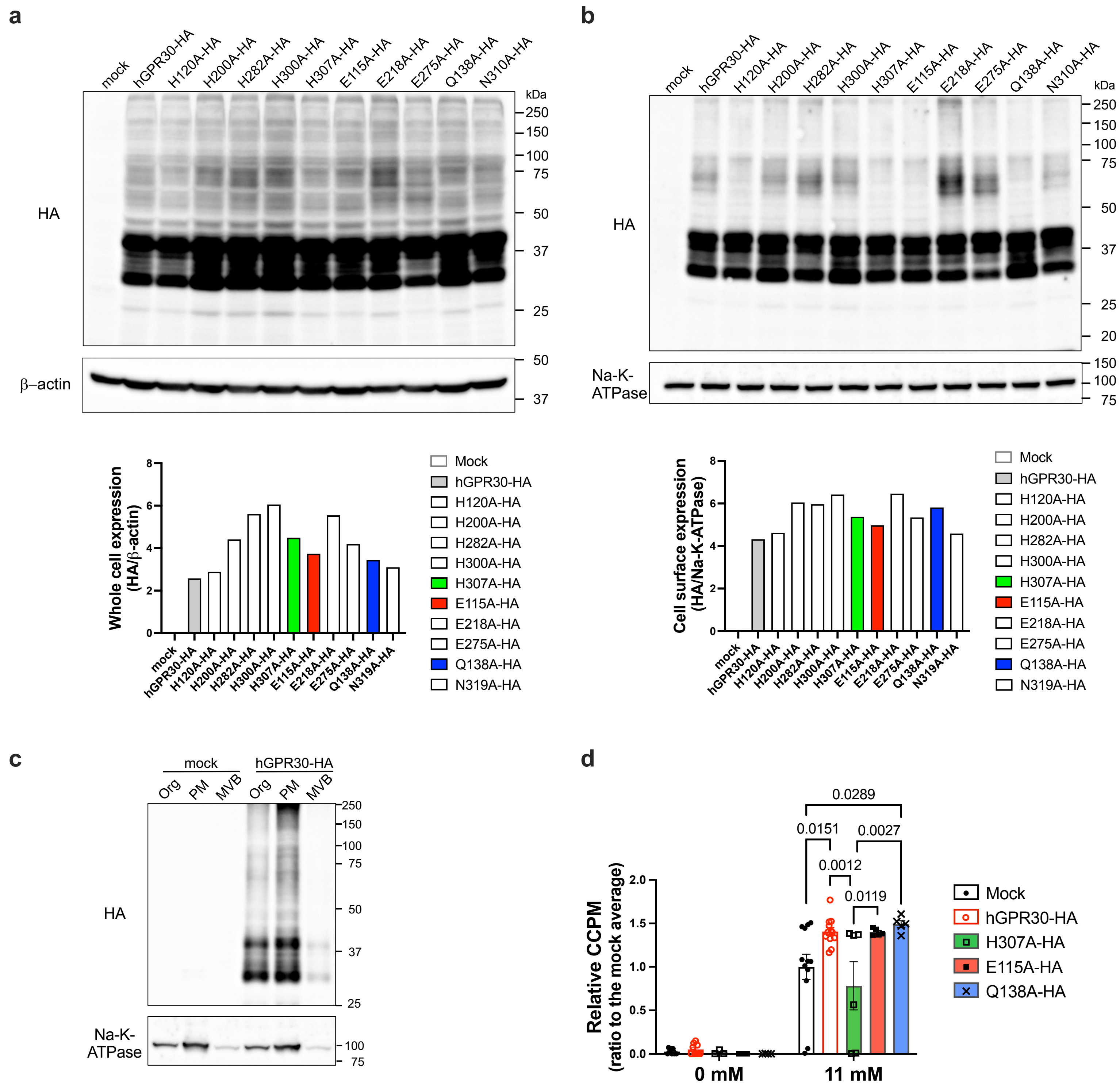

Supplementary Fig. 3. Cell surface expression and ligand binding of GPR30 mutants.

a) Whole cell expression of HA-tagged mutants analysed using western blotting. **b)** Cell surface expression of HA-tagged mutants, using cell surface biotinylation and avidin immunoprecipitation, analysed using western blotting. **c)** Expression of HA-tagged GPR30 in membrane fractions analysed using western blotting. Org, organellar membrane fraction including endoplasmic reticulum membrane; PM, plasma membrane fraction; MVB, multivesicular body fraction. **d)** Scintillation proximity assay (SPA) using the plasma membrane fraction incubated with sodium bicarbonate-[^{14}C] and SPA beads. The plasma membrane fractions of HEK293 cells transiently expressing mock, hGPR30-HA, E115A-HA, Q138A-HA, or H307A-HA were analysed. Statistical analysis: two-tailed unpaired t-test with Tukey's correction after two-way ANOVA (**d**). *P* values are shown if significant. Source data are provided as a Source Data file.

Supplementary Fig. 4

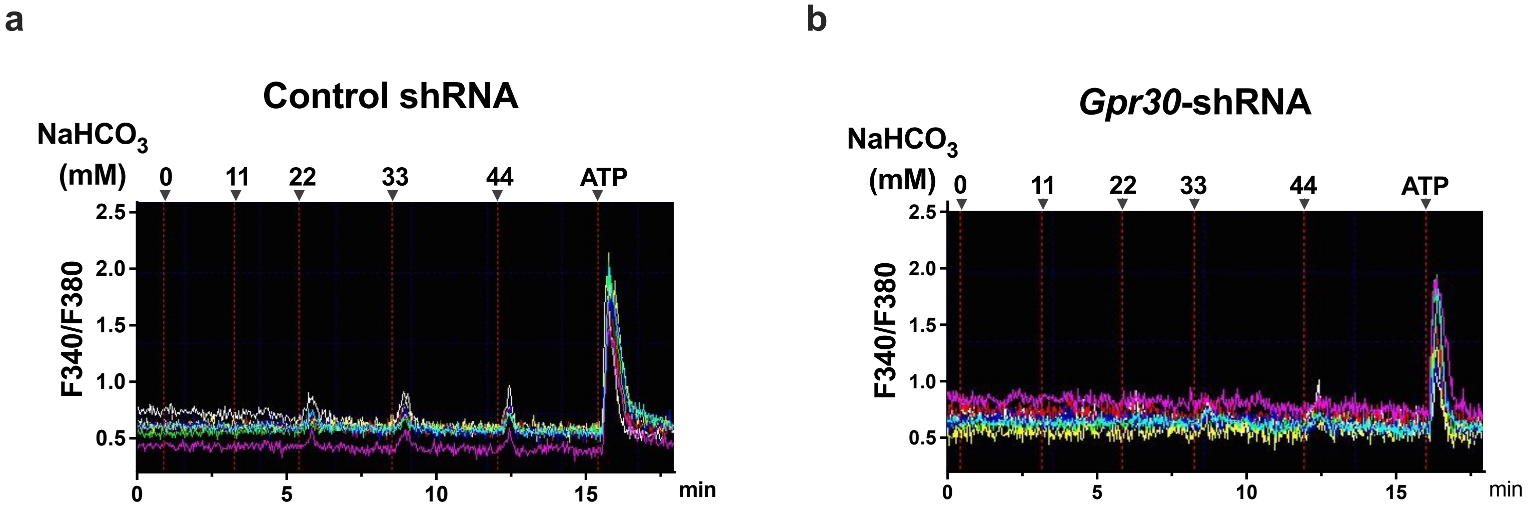

Supplementary Fig. 4. Endogenous GPR30 is activated by bicarbonate ions *in vitro*.

Intracellular calcium imaging using Fura-2-loaded mouse myoblast C2C12 cells stably expressing control shRNA (**a**) or *Gpr30*-shRNA (**b**), sequentially treated with indicated concentrations of sodium bicarbonate and ATP.

Supplementary Fig. 5

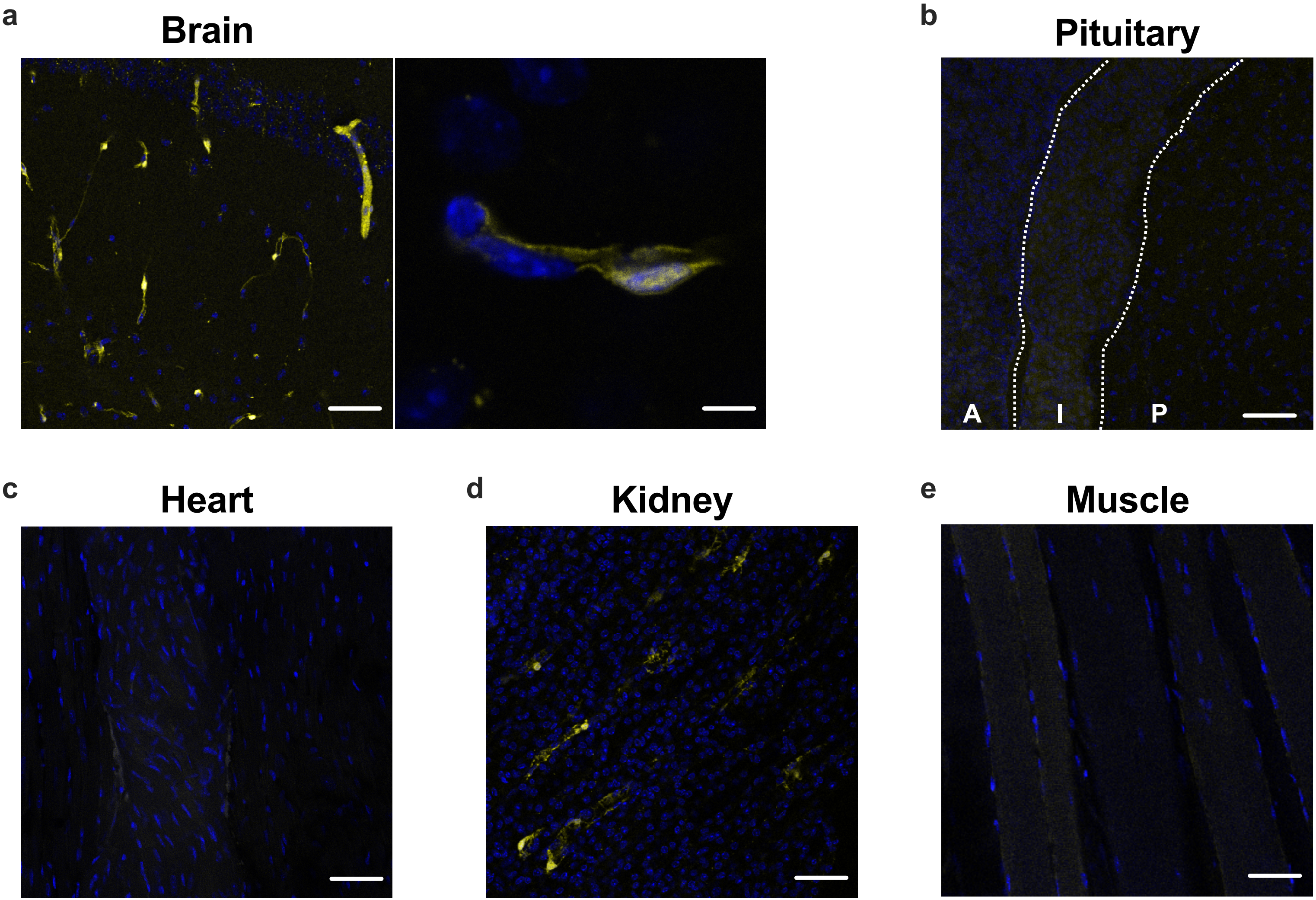

Supplementary Fig. 5. *Gpr30-Venus*-KI mouse reveals cell type-specific GPR30 expression in the brain and kidneys.

(a–e) Frozen sections of the brain (a), pituitary (b), heart (c), kidney (d), and muscle (e) obtained from heterozygous *Gpr30-Venus*-KI mice were analysed using confocal microscopy. In **(b)**, A, I, and P indicate the anterior, intermediate, and posterior lobes of the pituitary, respectively. The scale bar on the right panel of **(a)**, 5 μm. The other scale bars, 50 μm.

Supplementary Fig. 6

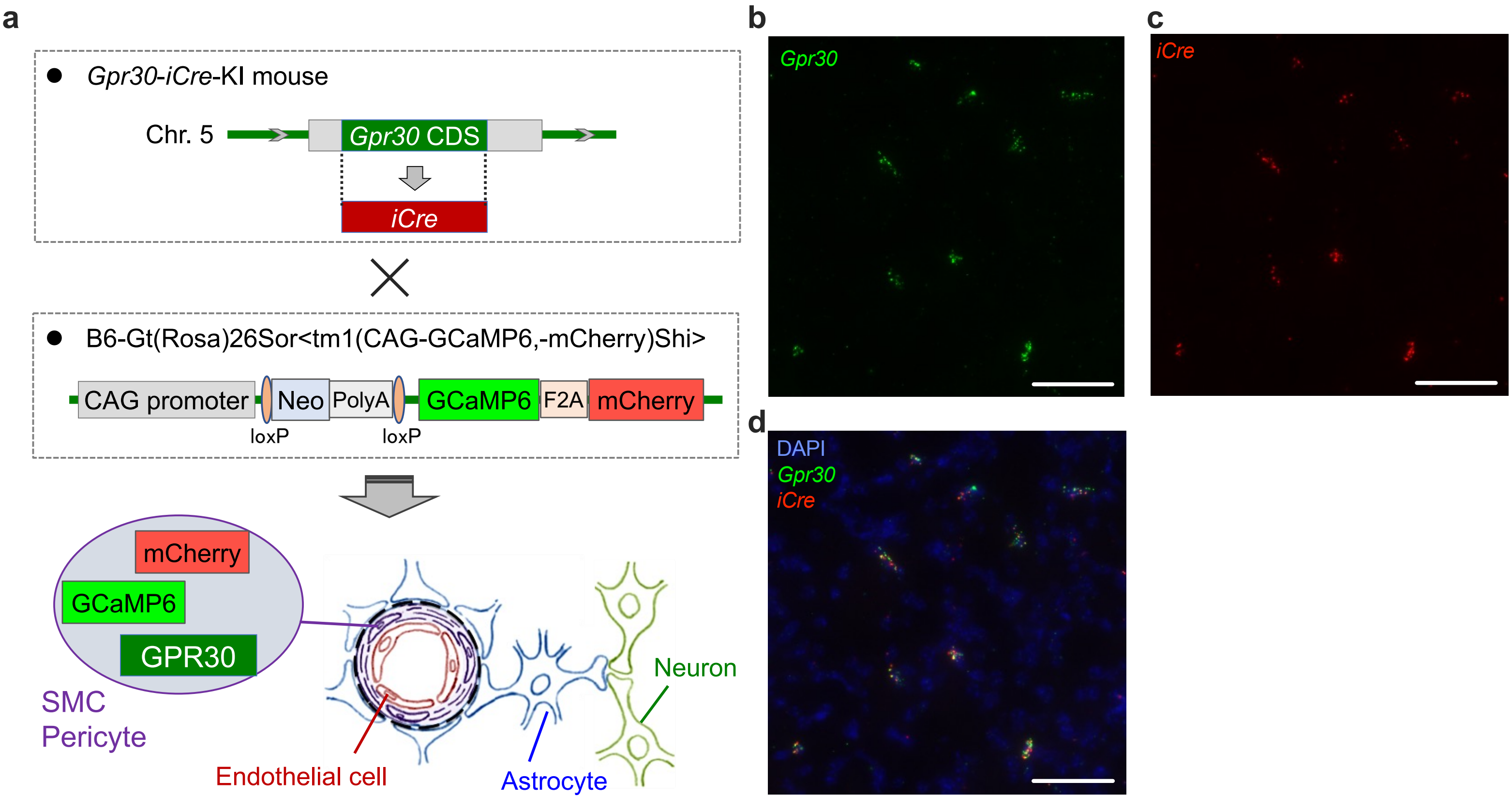

Supplementary Fig. 6. Generation of *Gpr30-iCre*-KI mouse to specifically analyse intracellular calcium levels in Gpr30-expressing cells.

a) Design of the *Gpr30*- codon-improved Cre recombinase (*iCre)* knock-in (*Gpr30-iCre*-KI) construct. The coding sequence of *Gpr30* was replaced in frame with that of *iCre*. The conditional GCaMP knock-in mice were generated by intercrossing *Gpr30-iCre*-KI mice and B6-Gt(Rosa)26Sor<tm1(CAG-GCaMP6,-mCherry)Shi>. **b–d)** Visualisation of *Gpr30* (**b, d**) and *iCre* (**c, d**) using multiple *in situ* hybridisation analyses of the *Gpr30-iCre*-KI brain cortex. Scale bars, 50 µm.

Supplementary Fig. 7

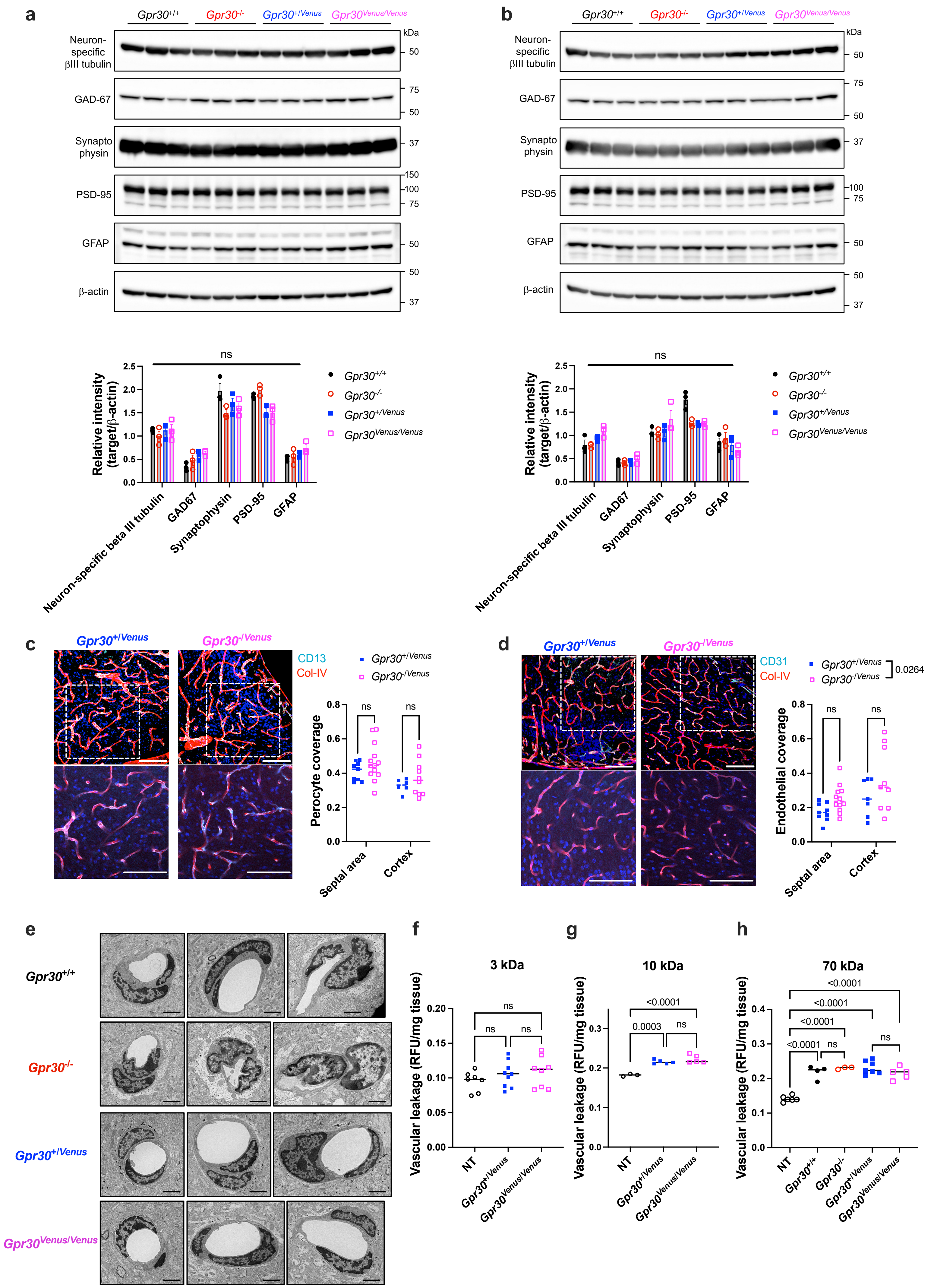

Supplementary Fig. 7. GPR30 deficiency does not impair the structural or functional integrity of the microvasculature at steady state.

a, b) Western blot analyses of neuronal (neuron-specific βIII tubulin, GAD-67), synaptic (synaptophysin and PSD-95), and astrocytic (GFAP) markers in the brain cortex (a) and hippocampus (b). For each target, all the samples were analysed on the same blot and normalized by reprobing the same membrane with anti-β-actin antibody. **c)** Quantification of pericyte coverage. **Left,** immunohistochemistry of CD13 (pericytes) and Col-IV (basement membrane) followed by 3D reconstructions of confocal image *z*-stacks of $Gpr30^{+/Venus}$ and $Gpr30^{-/Venus}$ brain slices. The lower images are the magnification of the white dashed square in the upper image. Scale bars, 100 µm. **Right,** pericyte coverage quantified as $CD13^{+}Col\text{-}IV^{+}$ volume/$Col\text{-}IV^{+}$ volume. **d)** Quantification of endothelial cell coverage. **Left,** immunohistochemistry of CD31 (endothelial cell) and Col-IV (basement membrane) followed by 3D reconstructions of confocal image *z*-stacks of $Gpr30^{+/Venus}$ and $Gpr30^{-/Venus}$ brain slices. The lower images are the magnification of the white dashed square in the upper image. Scale bars, 100 µm. **Right,** endothelial coverage quantified as $CD31^{+}Col\text{-}IV^{+}$ volume/$Col\text{-}IV^{+}$ volume. The endothelial coverage of $Gpr30^{+/Venus}$ and $Gpr30^{-/Venus}$ mice were significantly different. The difference was no longer significant when analysed in the septal area and cortex subgroups. **e)** Electron microscopy analysis of brain cortices of $Gpr30^{+/+}$, $Gpr30^{-/-}$, $Gpr30^{+/Venus}$, and $Gpr30^{Venus/Venus}$ mice. Scale bars, 2 µm. **f–h)** Mice were intravenously injected with 3 (f), 10 (g), or 70 kDa (h) dextran–Tetramethylrhodamine (TMR) and perfused with PBS after 3 h (f), 1 h (g), or 16 h (h). Vascular permeability was evaluated using the relative fluorescence units (RFU) of the leaked TMR per mg of wet tissue weight. NT indicates mice that were

not injected with dextran–TMR. Statistical analysis: two-tailed unpaired t-test with Bonferroni's correction after two-way ANOVA (**a–d)**. Two-tailed unpaired t-test with Bonferroni's correction after one-way ANOVA (**f–h)**. Data are presented as mean values ± SEM. *P* values are shown if significant. ns indicates no significant difference. Source data are provided as a Source Data file.

Supplementary Fig. 8

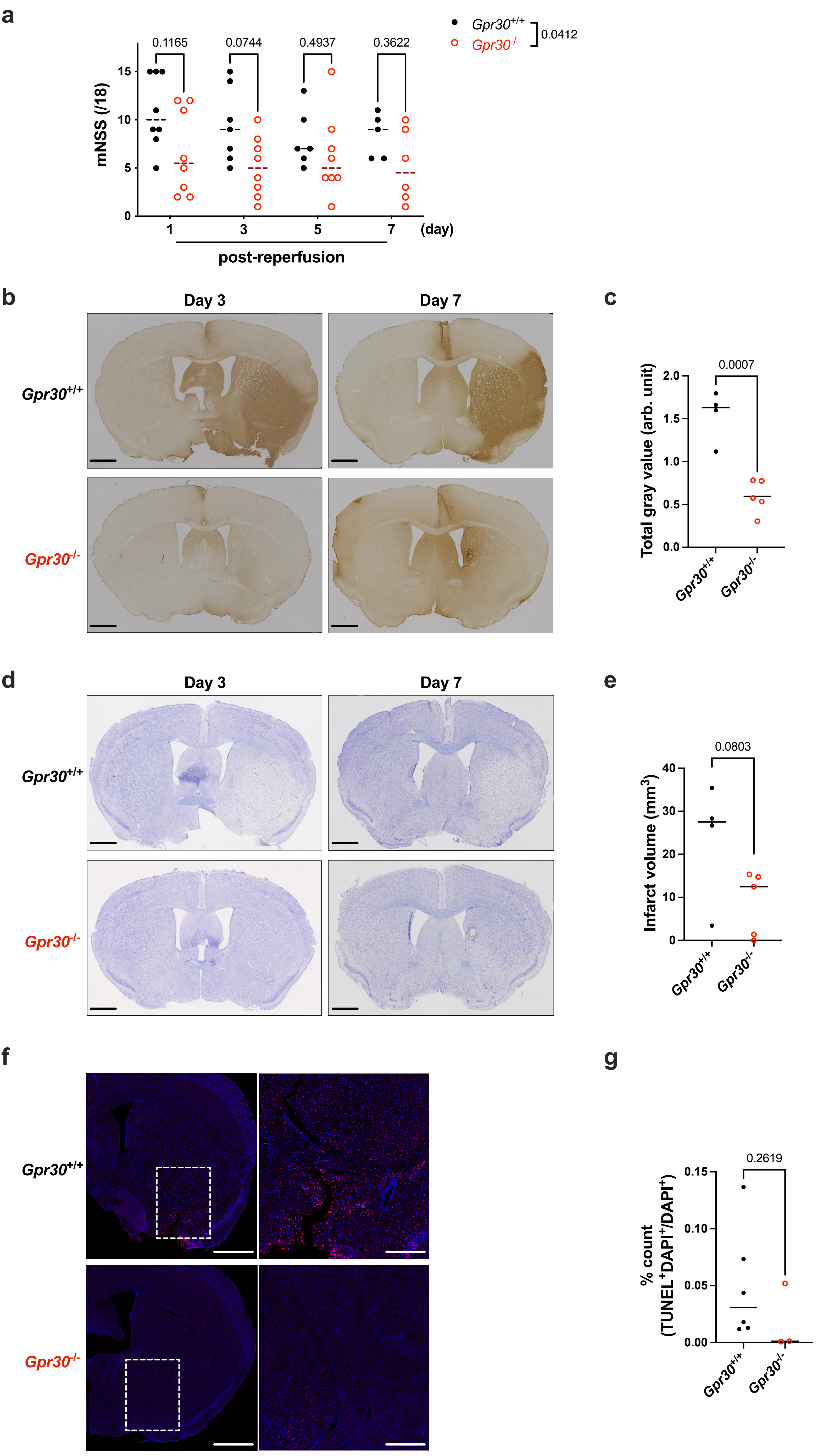

Supplementary Fig. 8. GPR30-deficient mice are resistant to ischaemia-reperfusion injury at 3 and 7 days after MCAO.

a) Modified Neurological Severity Score (mNSS) of *Gpr30*$^{+/+}$ and *Gpr30*$^{-/-}$ mice 1 to 7 days after reperfusion. **b, c)** Evaluation of the blood–brain barrier (BBB) impairment. Representative images of IgG immunostaining of *Gpr30*$^{+/+}$ and *Gpr30*$^{-/-}$ mouse brains at 3 and 7 days after MCAO (b) and quantification of IgG staining of whole brain sections 7 days after MCAO (c). Scale bars, 1 mm. **d, e)** Evaluation of infarct volume. Representative images of cresyl violet staining of *Gpr30*$^{+/+}$ and *Gpr30*$^{-/-}$ mouse brains 3 and 7 days after MCAO (d) and quantification of cresyl violet staining of whole brain sections 7 days after MCAO (e). Scale bars, 1 mm. **f, g)** Evaluation of apoptosis using TUNEL staining of whole brain sections 3 days after MCAO. Representative images of TUNEL staining (f) show that TUNEL-positive apoptotic cells were hardly detectable in *Gpr30*$^{-/-}$ mice. The right images are the magnification of the white dashed square in the left images. Scale bars, 1 mm (the left) and 330 µm (the right). Statistical analysis: two-tailed mixed-effects analysis with Bonferroni's correction (**a**). Two-tailed unpaired t-test (**c, e**). Two tailed Mann–Whitney test (**g**). Data are presented as dot plots with medians. All *p* values are shown. Source data are provided as a Source Data file.

Supplementary Fig. 9

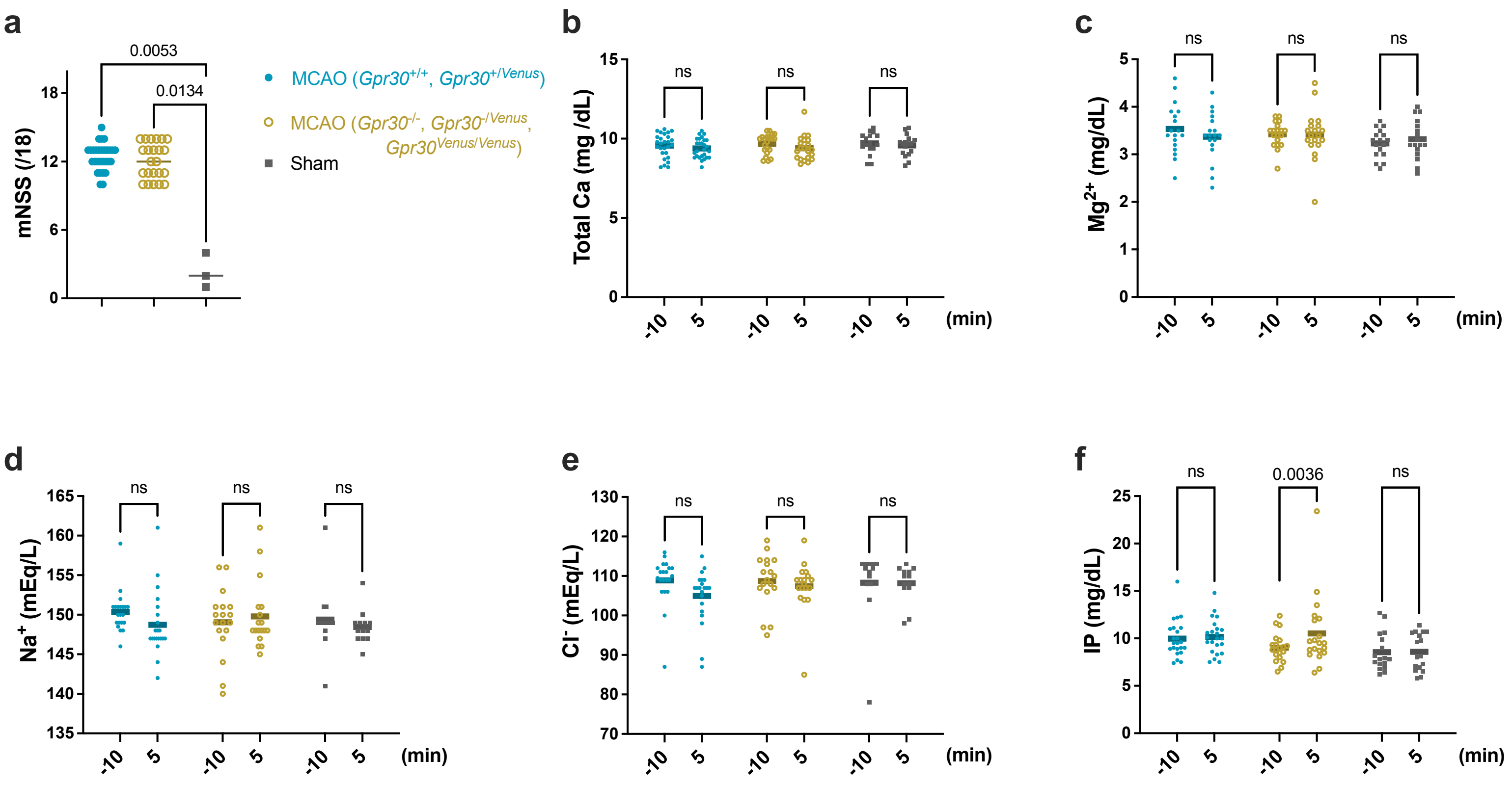

Supplementary Fig. 9. Serum electrolyte concentrations before and after reperfusion.

a) The modified Neurological Severity Score (mNSS) was analysed 45 min after MCA occlusion. **b–f)** Blood was collected from the facial vein 10 min before and 5 min after reperfusion. Serum concentrations of total Ca (b), Mg^{2+} (c), Na^{+} (d), Cl^{-} (e), and inorganic phosphorus (IP, f) were measured. Statistical analysis: **a)** Two-tailed Kruskal–Wallis test with Dunn's correction. Data are presented as dot plots with medians. **b–f)** Two-tailed unpaired t-test with Bonferroni's correction after repeated measures two-way ANOVA. Data are presented as dot plots with mean values. *P* values are shown if significant. ns indicates no significant difference. Source data are provided as a Source Data file.

Supplementary Fig. 10

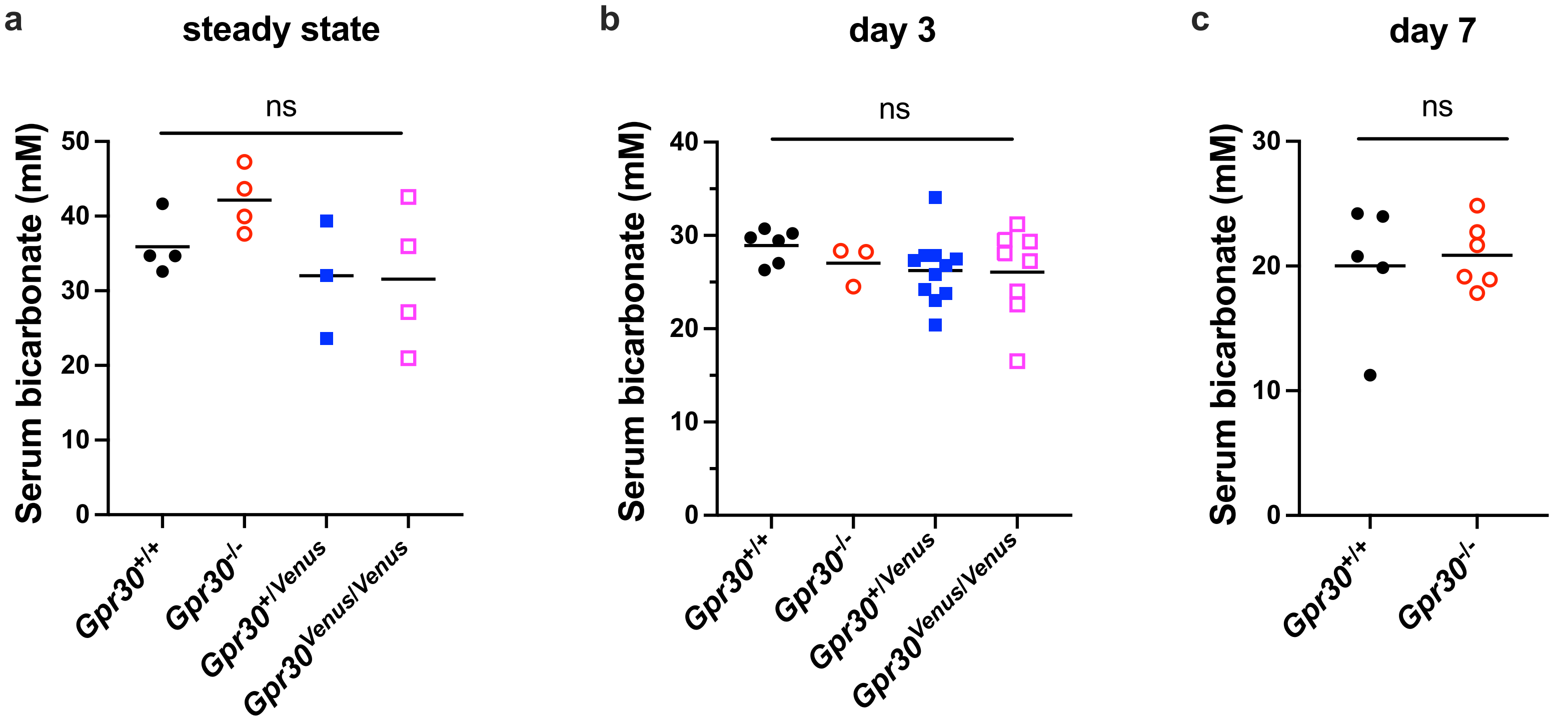

Supplementary Fig. 10. The serum bicarbonate concentrations at steady state and 3 and 7 days after reperfusion.

a–c) Serum bicarbonate levels were comparable in *Gpr30*$^{+/+}$, *Gpr30*$^{-/-}$, *Gpr30*$^{+/Venus}$, and *Gpr30*$^{Venus/Venus}$ mice at steady state (a), 3 (b), and 7 days (c) after MCAO. Statistical analysis: ns indicates no significant main effect using one-way ANOVA (**a, b**) and two-tailed unpaired t-test (**c**). Data are presented as dot plots with mean values. Source data are provided as a Source Data file.

Supplementary Table 1. The components and pH of all the buffers used to test functional effects of bicarbonate.

The components of Hank's Balanced Salt Solution (HBSS) used in this study	
components	Concentration (mM)
NaCl	136.99
KCl	5.36
KH_2PO_4	0.44
Na_2HPO_4	0.34
D-Glucose	5.55
$CaCl_2$	1.26
$MgCl_2$	0.49
$MgSO_4$	0.41
HEPES	5.0
The pH of the buffers used to test functional effects of bicarbonate in this study	
Assay	Buffer and pH
TGFα shedding assay	HBSS: 7.4<br>alkaline phosphatase (AP) solution: 9.4
Calcium assay	HBSS: 7.4
Cyclic AMP assay	HBSS: 7.4
Inositol phosphate accumulation assay	HBSS: 7.4
SPA	50 mM HEPES, 5 mM $MgCl_2$, 1 mM $CaCl_2$, 100 mM NaCl, 5% BSA, pH 7.5

Supplementary Table 2. Target position and sequence of shRNA

(Methods: *shRNA vector and knockdown cell line*)

shRNA	Position NM_029771	Sequence (5′ → 3′)
#1	83–102	sense: GATCCCC-(TCTGGCCCTCAACTTGTCC)-TTCAAGAGA-(GGACAAGTTGAGGGCCAGA)-TTTTTGGAAA<br>antisense: AGCTTTTCCAAAAA-(TCTGGCCCTCAACTTGTCC)-TCTCTTGAA-(GGACAAGTTGAGGGCCAGA)-GGG
#2	84–103	sense: GATCCCC-(CTGGCCCTCAACTTGTCCC)-TTCAAGAGA-(GGGACAAGTTGAGGGCCAG)-TTTTTGGAAA<br>antisense: AGCTTTTCCAAAAA-(CTGGCCCTCAACTTGTCCC)-TCTCTTGAA-(GGGACAAGTTGAGGGCCAG)-GGG
#3	163–182	sense: GATCCCC-(ACGTGATTGCCCTCTTCCT)-TTCAAGAGA-(AGGAAGAGGGCAATCACGT)-TTTTTGGAAA<br>antisense: AGCTTTTCCAAAAA-(ACGTGATTGCCCTCTTCCT)-TCTCTTGAA-(AGGAAGAGGGCAATCACGT)-GGG
#4	1523–1543	sense: GATCCCC-(ACAGGCCACATAGTCAACCTTCTCGAGAAGGTTGACTATGTGGCCTGT)-TTTTTGGAAA<br>antisense: AGCTTTTCCAAAAA-(ACAGGCCACATAGTCAACCTTCTCGAGAAGGTTGACTATGTGGCCTGT)-GGG
#5	1670–1690	sense: GATCCCC-(GCCACGCTCAAGGCCGTCATTCTCGAGAATGACGGCCTTGAGCGTGGC)-TTTTTGGAAA<br>antisense: AGCTTTTCCAAAAA-(GCCACGCTCAAGGCCGTCATTCTCGAGAATGACGGCCTTGAGCGTGGC)-GGG
#6	761–781	sense: GATCCCC-(GAGCATCAGCAGTACGTGATTCTCGAGAATCACGTACTGCTGATGCTC)-TTTTTGGAAA<br>antisense: AGCTTTTCCAAAAA-(GAGCATCAGCAGTACGTGATTCTCGAGAATCACGTACTGCTGATGCTC)-GGG
#7	1426–1446	sense: GATCCCC-(GCTGCCGGAGAACGTCTTCATCTCGAGATGAAGACGTTCTCCGGCAGC)-TTTTTGGAAA<br>antisense: AGCTTTTCCAAAAA-(GCTGCCGGAGAACGTCTTCATCTCGAGATGAAGACGTTCTCCGGCAGC)-GGG
#8	807–825	sense: GATCCCC-(ACCATCTTCCTCTTTCCTATTG)-TTCAAGAGA-(CAATAGGAAAGAGGAAGATGGT)-TTTTTGGAAA<br>antisense: AGCTTTTCCAAAAA-(ACCATCTTCCTCTTTCCTATTG)-TCTCTTGAA-(CAATAGGAAAGAGGAAGATGGT)-GGG

Supplementary Table 3. Primer sequence

(Methods: *RNA isolation and quantitative PCR*)

Gene	Sequence (5′ → 3′)
Tubb3	Forward: GTCTCTAGCCGCGTGAAGTC<br>Reverse: CATCGCTGATGACCTCCCAG
Gad1	Forward: CCAGCACGTACTCCTGTGAC<br>Reverse: GGCTACGCCACACCAAGTAT
Gfap	Forward: TTGCTGGAGGGCGAAGAAAA<br>Reverse: TGGTGAGCCTGTATTGGGAC
Pdgfra	Forward: CGAAAAATTGTGTCCACCGGG<br>Reverse: CAGCGTGGTGTAGAGGTTGT
Mbp	Forward: AAAGAAGAGAAGCGTGGGCA<br>Reverse: TGTGCTTGGAGTCTGTCACC
Pdgfrb	Forward: GTGGAGATTCGCAGGAGGTC<br>Reverse: ATAGCGTGGCTTCTTCTGCC
Cldn5	Forward: AGTTAAGGCACGGGTAGCAC<br>Reverse: GTACTTCTGTGACACCGGCA
Abcc9	Forward: GGAGCTGACAGACACGAACA<br>Reverse: GGTCGGCCAAGTTCCTTACA
Tie2	Forward: TGTGAAGGTCGAGTTCGAGG<br>Reverse: CTGAGTGGATGAAGGAGCCATT
Gpr30 (1)	Forward: AACCTCACTGGGGACCTCTC<br>Reverse: CGGAAGCTGATGTTCACCAC
Gpr30 (2)	Forward: CCATGCACCCACCAAAACAGC<br>Reverse: AGAAAACCAGAAGGGTGGACAG
Actb	Forward: CATCCGTAAAGACCTCTATGCCAAC<br>Reverse: ATGGAGCCACCGATCCACA

Supplementary Table 4. The primary/secondary antibodies used in this study, along the validation statements.

Antibody (clone name); supplier name, catalog number	validation statement
phospho-p44/42 MAPK (Erk1/2); Cell Signaling Technology, #9101	https://www.cellsignal.com/products/primary-antibodies/phospho-p44-42-mapk-erk1-2-thr202-tyr204-antibody/9101?country=JP&language=en
ERK2 (C-14); Santa Cruz Biotechnology, sc-154	https://www.scbt.com/p/erk-2-antibody-c-14
anti-HA High Affinity (3F10); Roche, 11867423001	https://www.sigmaaldrich.com/JP/en/product/roche/roahaha
Na-K-ATPase; Cell Signaling Technology, #3010	https://www.cellsignal.com/products/primary-antibodies/na-k-atpase-antibody/3010?country=JP&language=en
β-Actin (AC-15); Santa Cruz Biotechnology, sc-69879	https://www.scbt.com/p/beta-actin-antibody-ac-15?requestFrom=search&bvstate=pg:2/ct:r
PSD95 (EPR23124-118); Abcam, ab238135	https://www.abcam.com/en-mx/products/primary-antibodies/psd95-antibody-epr23124-118-synaptic-marker-ab238135
Synaptophysin (YE269); Abcam, ab32127	https://www.abcam.com/en-th/products/primary-antibodies/synaptophysin-antibody-ye269-ab32127
GAD67 (K-87); Abcam, ab26116	https://www.abcam.com/en-fi/products/primary-antibodies/gad1-gad67-antibody-k-87-ab26116
Neuron-specific beta -III Tubulin (TuJ-1); R&D systems, MAB1195	https://www.rndsystems.com/products/neuron-specific-beta-iii-tubulin-antibody-tuj-1_mab1195
GFAP; Proteintech, 16825-I-AP	https://www.ptglab.com/products/GFAP-Antibody-16825-1-AP.htm
anti-mouse collagen IV; Bio-Rad, #2150-1470	https://www.bio-rad-antibodies.com/polyclonal/mouse-collagen-iv-antibody-2150-1470.html?f=purified
anti-CD13-Alexa 647; BD Biosciences, #564352	https://www.bdbiosciences.com/en-eu/products/reagents/flow-cytometry-reagents/research-reagents/single-color-antibodies-ruo/alexa-fluor-647-rat-anti-mouse-cd13.564352

anti-CD31-Alexa 647; BioLegend, #102515	https://www.biolegend.com/en-us/products/alexa-fluor-647-anti-mouse-cd31-antibody-3094?GroupID=BLG10531
anti-rabbit IgG, HRP-linked Antibody; Cell Signaling Technology, #7074	https://www.cellsignal.com/products/secondary-antibodies/anti-rabbit-igg-hrp-linked-antibody/7074
anti-mouse IgG, HRP-linked Antibody; Cell Signaling Technology, #7076	https://www.cellsignal.com/products/secondary-antibodies/anti-mouse-igg-hrp-linked-antibody/7076
anti-Rat IgG, HRP-Linked Whole Ab Goat; Cytiva, NA935	https://www.citeab.com/antibodies/3288278-na935-amersham-ecl-rat-igg-hrp-linked-whole-antibod
Alexa 546-labelled donkey anti-rabbit IgG; Invitrogen, A10040	https://www.citeab.com/antibodies/2401390-a10040-donkey-anti-rabbit-igg-h-l-highly-cross-ads.

Supplementary Table 5. Biological and technical replicates in *in vitro* experiments.

Figure/Panel	Biological replicates	Technical replicates per experiment	Number of independent experiments	memo
Fig. 1a	2	4	3	
Fig. 1b	11	4	6	
Fig. 1c	1	4	1	
Fig. 1d	1	4	1	
Fig. 1e	3	4	9	
Fig. 1f	1	4	1	TGFα shedding assay with similar results was repeated 10 times.
Fig. 2a	2	4	4	
Fig. 2b	1	1	1	
Fig. 2c	1	3	3	
Fig. 2d	2	4	4	
Fig. 2e	7	1	8	
Fig. 2f	2	1	2	
Fig. 2g	5	1	5	
Fig. 2h	1	3	4	
Fig. 2i	5	3	5	
Fig. 3c	3	4	3	
Fig. 3d	4	3	4	
Fig. 3g	3	1	3	
Fig. 3h	1	4	3	

Fig. 4a, b	1	1	6	391 cells examined over 6 experiments.
Fig. 4c	1	3	3	
Fig. 4d	1	1	11/10 (control/shRNA)	865/784 cells examined over 11/10 experiments.
Supplementary Fig. 2a	6	3	6	
Supplementary Fig. 2b	2	1	2	
Supplementary Fig. 2c	1	4	1	
Supplementary Fig. 2d	2	5	2	
Supplementary Fig. 2e	2	4	2	
Supplementary Fig. 2f	1	4	1	
Supplementary Fig. 2g	1	4	1	
Supplementary Fig. 2h	1	4	1	The result was confirmed by TGFα shedding assay.
Supplementary Fig. 2i	1	3	1	TGFα shedding assay with similar results was repeated twice.
Supplementary Fig. 2j	1	4	1	
Supplementary Fig. 2k	1	3	1	
Supplementary Fig. 2l	1	3	1	
Supplementary Fig. 3a	1	1	2	
Supplementary Fig. 3b	1	1	2	
Supplementary Fig. 3c	4	1	4	
Supplementary Fig. 3d	3–8	2	8	
Supplementary Fig. 4a	1	1	11	865 cells examined over 11 experiments.

Supplementary Fig. 4b	1	1	10	784 cells examined over 10 experiments.

Supplementary Table 6. Species, strain, substrain, sex, age, genotype, and number of animals in *in vivo* experiments.

Mice were used in all experiments.

Figure/Panel	strain /substrain	sex	age	genotype	number
Fig. 5b	C57BL/6J (The Jackson Laboratory)	male	8	$Gpr30^{+/Venus}$	3
		male	8	$Gpr30^{-/Venus}$	3
Fig. 5c–d	C57BL/6J (The Jackson Laboratory)	male	8	$Gpr30^{+/Venus}$	3
		male	15	$Gpr30^{+/Venus}$	1
Fig. 5e	C57BL/6J (The Jackson Laboratory)	female	6	$Gpr30^{+/+}$	2
		female	8	$Gpr30^{+/+}$	2
		male	9	$Gpr30^{+/+}$	3
		male	9	$Gpr30^{-/-}$	2
		male	10	$Gpr30^{+/+}$	3
		male	10	$Gpr30^{-/-}$	1
Fig. 5f	C57BL/6J (The Jackson Laboratory)	male	2	$Gpr30^{+/+}$	1
		male	6	$Gpr30^{+/+}$	1
		male	25	$Gpr30^{+/+}$	1
Fig. 5g	C57BL/6J (The Jackson Laboratory)	male	6	$Gpr30^{+/+}$	2
		male	6	$Gpr30^{-/-}$	1
Fig. 5h, i	C57BL/6J (The Jackson Laboratory)	male	3	$Gpr30^{+/+}$	2
		female	4	$Gpr30^{+/+}$	2
		male	5	$Gpr30^{+/+}$	2
Fig. 6a–c	C57BL/6J (The Jackson Laboratory)	female	2	$Gpr30^{+/Venus}$	1
		female	2	$Gpr30^{Venus/Venus}$	2
		female	3	$Gpr30^{+/Venus}$	2
		female	3	$Gpr30^{Venus/Venus}$	1

		female	4	$Gpr30^{+/Venus}$	5
		male	4	$Gpr30^{+/Venus}$	1
		female	4	$Gpr30^{Venus/Venus}$	5
		male	5	$Gpr30^{+/Venus}$	2
		female	5	$Gpr30^{Venus/Venus}$	1
		male	5	$Gpr30^{Venus/Venus}$	3
Fig. 6d–f	C57BL/6J (C57BL/6JJ msSlc)	male	9	$Gpr30^{+/iCre}$; Rosa26-GCaMP6	1
		female	11	$Gpr30^{+/iCre}$; Rosa26-GCaMP6	2
		female	12	$Gpr30^{iCre/iCre}$; Rosa26-GCaMP6	1
		male	20	$Gpr30^{+/iCre}$; Rosa26-GCaMP6	1
		male	21	$Gpr30^{iCre/iCre}$; Rosa26-GCaMP6	1
		male	25	$Gpr30^{+/iCre}$; Rosa26-GCaMP6	1
		male	30	$Gpr30^{+/iCre}$; Rosa26-GCaMP6	1
		male	30	$Gpr30^{iCre/iCre}$; Rosa26-GCaMP6	2
Fig. 6g–i	C57BL/6J (C57BL/6JJ msSlc)	male	9	$Gpr30^{+/iCre}$; Rosa26-GCaMP6	1
		female	11	$Gpr30^{+/iCre}$; Rosa26-GCaMP6	2
		female	12	$Gpr30^{iCre/iCre}$; Rosa26-GCaMP6	1
		male	20	$Gpr30^{+/iCre}$; Rosa26-GCaMP6	1
		male	21	$Gpr30^{iCre/iCre}$; Rosa26-GCaMP6	1
Fig. 7b	C57BL/6J (The Jackson Laboratory)	male	14	$Gpr30^{+/Venus}$	5
		male	14	$Gpr30^{Venus/Venus}$	4
		male	15	$Gpr30^{+/Venus}$	3
		male	15	$Gpr30^{Venus/Venus}$	2
		male	16	$Gpr30^{+/Venus}$	3
		male	16	$Gpr30^{Venus/Venus}$	2
Fig. 7c–d	C57BL/6J (The Jackson Laboratory)	male	14	$Gpr30^{+/Venus}$	4
		male	14	$Gpr30^{Venus/Venus}$	3
		male	15	$Gpr30^{+/Venus}$	3
		male	15	$Gpr30^{Venus/Venus}$	2
		male	16	$Gpr30^{+/Venus}$	3
		male	16	$Gpr30^{Venus/Venus}$	2
Fig. 7e–f	C57BL/6J (The	male	14	$Gpr30^{+/Venus}$	5
		male	14	$Gpr30^{Venus/Venus}$	4
		male	15	$Gpr30^{+/Venus}$	3

	Jackson Laboratory)	male	15	*Gpr30*$^{Venus/Venus}$	2
		male	16	*Gpr30*$^{+/Venus}$	3
		male	16	*Gpr30*$^{Venus/Venus}$	2
Fig. 7g–h	C57BL/6J (The Jackson Laboratory)	male	14	*Gpr30*$^{+/Venus}$	4
		male	14	*Gpr30*$^{Venus/Venus}$	3
		male	15	*Gpr30*$^{+/Venus}$	3
		male	15	*Gpr30*$^{Venus/Venus}$	2
		male	16	*Gpr30*$^{+/Venus}$	3
		male	16	*Gpr30*$^{Venus/Venus}$	2
Fig. 8b	C57BL/6J (The Jackson Laboratory)	male	10–20	*Gpr30*$^{+/+}$, *Gpr30*$^{+/Venus}$	27
		male	10–20	*Gpr30*$^{–/–}$, *Gpr30*$^{–/Venus}$, *Gpr30*$^{Venus/Venus}$	21
		male	10–18	sham	23
Fig. 8d–e	C57BL/6J (The Jackson Laboratory)	male	9	*Gpr30*$^{+/+}$	1
		male	9	*Gpr30*$^{–/–}$	1
		male	10	*Gpr30*$^{+/+}$	4
		male	10	*Gpr30*$^{–/–}$	3
		male	10	*Gpr30*$^{+/Venus}$	1
		male	11	*Gpr30*$^{Venus/Venus}$	1
		male	12	*Gpr30*$^{+/Venus}$	3
		male	12	*Gpr30*$^{Venus/Venus}$	2
		male	13	*Gpr30*$^{+/Venus}$	1
		male	13	*Gpr30*$^{Venus/Venus}$	2
		male	14	*Gpr30*$^{–/–}$	2
		male	15	*Gpr30*$^{Venus/Venus}$	1
Fig. 8g–h	C57BL/6J (The Jackson Laboratory)	male	11	*Gpr30*$^{+/+}$	1
		male	11	*Gpr30*$^{–/–}$	2
		male	12	*Gpr30*$^{+/+}$	1
		male	13	*Gpr30*$^{+/+}$	2
		male	14	*Gpr30*$^{Venus/Venus}$	1
		male	17	*Gpr30*$^{Venus/Venus}$	1
		male	20	*Gpr30*$^{+/Venus}$	2
		male	20	*Gpr30*$^{Venus/Venus}$	1
		male	21	*Gpr30*$^{+/Venus}$	1

		male	21	$Gpr30^{Venus/Venus}$	1
		male	23	$Gpr30^{+/+}$	1
		male	23	$Gpr30^{-/-}$	1
Supplementary Fig. 5	C57BL/6J (The Jackson Laboratory)	male	25	$Gpr30^{+/Venus}$	1
Supplementary Fig. 6b–d	C57BL/6J (C57BL/6JJmsSlc)	female	3	$Gpr30^{+/iCre}$	4
		male	3	$Gpr30^{+/iCre}$	2
		female	19	$Gpr30^{+/iCre}$	2
		female	19	$Gpr30^{iCre/iCre}$	2
Supplementary Fig. 7a–b	C57BL/6J (The Jackson Laboratory)	male	15–17	$Gpr30^{+/+}$	3
		male	15–17	$Gpr30^{-/-}$	3
		male	15–17	$Gpr30^{+/Venus}$	3
		male	15–17	$Gpr30^{Venus/Venus}$	3
Supplementary Fig. 7c	C57BL/6J (The Jackson Laboratory)	male	8	$Gpr30^{+/Venus}$	3
		male	8	$Gpr30^{-/Venus}$	4
Supplementary Fig. 7d	C57BL/6J (The Jackson Laboratory)	male	8	$Gpr30^{+/Venus}$	3
		male	8	$Gpr30^{-/Venus}$	4
Supplementary Fig. 7e	C57BL/6J (The Jackson Laboratory)	male	9	$Gpr30^{+/Venus}$	3
		male	9	$Gpr30^{Venus/Venus}$	3
		male	11	$Gpr30^{+/+}$	1
		male	11	$Gpr30^{-/-}$	1
		male	15	$Gpr30^{+/+}$	1
		male	15	$Gpr30^{-/-}$	1
		male	18	$Gpr30^{+/+}$	1
		male	18	$Gpr30^{-/-}$	1
Supplementary Fig. 7f	C57BL/6J (The Jackson Laboratory)	male	11	$Gpr30^{+/+}$	3
		male	11	$Gpr30^{+/Venus}$	3
		male	11	$Gpr30^{Venus/Venus}$	3
		male	14	$Gpr30^{+/+}$	1

		male	14	*Gpr30*$^{+/Venus}$	2
		male	14	*Gpr30*$^{Venus/Venus}$	2
		male	16	*Gpr30*$^{+/Venus}$	1
		male	16	*Gpr30*$^{Venus/Venus}$	1
		male	18	*Gpr30*$^{+/Venus}$	2
		male	18	*Gpr30*$^{Venus/Venus}$	1
		male	20	*Gpr30*$^{+/Venus}$	3
		male	20	*Gpr30*$^{Venus/Venus}$	2
Supplementary Fig. 7g	C57BL/6J (The Jackson Laboratory)	male	7	*Gpr30*$^{+/+}$	2
		male	7	*Gpr30*$^{+/Venus}$	6
		male	7	*Gpr30*$^{Venus/Venus}$	5
Supplementary Fig. 7h	C57BL/6J (The Jackson Laboratory)	male	5	*Gpr30*$^{+/-}$	1
		male	7	*Gpr30*$^{+/Venus}$	2
		male	7	*Gpr30*$^{Venus/Venus}$	1
		male	8	*Gpr30*$^{+/+}$	1
		male	8	*Gpr30*$^{-/-}$	1
		male	8	*Gpr30*$^{+/Venus}$	3
		male	8	*Gpr30*$^{Venus/Venus}$	3
		male	9	*Gpr30*$^{+/+}$	2
		male	9	*Gpr30*$^{-/-}$	1
		male	9	*Gpr30*$^{+/Venus}$	4
		male	9	*Gpr30*$^{Venus/Venus}$	2
		male	10	*Gpr30*$^{+/+}$	2
		male	12	*Gpr30*$^{+/+}$	1
		male	12	*Gpr30*$^{-/-}$	1
Supplementary Fig. 8a	C57BL/6J (The Jackson Laboratory)	male	14	*Gpr30*$^{+/+}$	3
		male	14	*Gpr30*$^{-/-}$	2
		male	15	*Gpr30*$^{+/+}$	1
		male	15	*Gpr30*$^{-/-}$	1
		male	18	*Gpr30*$^{+/+}$	3
		male	18	*Gpr30*$^{-/-}$	4
		male	20	*Gpr30*$^{+/+}$	1
		male	20	*Gpr30*$^{-/-}$	1

Supplementary Fig. 8b–g	C57BL/6J (The Jackson Laboratory)	male	14	*Gpr30*$^{+/+}$ (3-day model)	6
		male	14	*Gpr30*$^{-/-}$ (3-day model)	3
		male	15	*Gpr30*$^{+/+}$ (7-day model)	1
		male	15	*Gpr30*$^{-/-}$ (7-day model)	1
		male	18	*Gpr30*$^{+/+}$ (7-day model)	2
		male	18	*Gpr30*$^{-/-}$ (7-day model)	3
		male	20	*Gpr30*$^{+/+}$ (7-day model)	1
		male	20	*Gpr30*$^{-/-}$ (7-day model)	1
Supplementary Fig. 9a	C57BL/6J (The Jackson Laboratory)	male	10–20	*Gpr30*$^{+/+}$, *Gpr30*$^{+/Venus}$	29
		male	10–20	*Gpr30*$^{-/-}$, *Gpr30*$^{-/Venus}$, *Gpr30*$^{Venus/Venus}$	23
		male	10–18	sham	3
Supplementary Fig. 9b	C57BL/6J (The Jackson Laboratory)	male	10–20	*Gpr30*$^{+/+}$, *Gpr30*$^{+/Venus}$	27
		male	10–20	*Gpr30*$^{-/-}$, *Gpr30*$^{-/Venus}$, *Gpr30*$^{Venus/Venus}$	21
		male	10–18	sham	17
Supplementary Fig. 9c	C57BL/6J (The Jackson Laboratory)	male	10–20	*Gpr30*$^{+/+}$, *Gpr30*$^{+/Venus}$	18
		male	10–20	*Gpr30*$^{-/-}$, *Gpr30*$^{-/Venus}$, *Gpr30*$^{Venus/Venus}$	19
		male	10–18	sham	17
Supplementary Fig. 9d	C57BL/6J (The Jackson Laboratory)	male	10–20	*Gpr30*$^{+/+}$, *Gpr30*$^{+/Venus}$	21
		male	10–20	*Gpr30*$^{-/-}$, *Gpr30*$^{-/Venus}$, *Gpr30*$^{Venus/Venus}$	18
		male	10-18	sham	13
Supplementary Fig. 9e	C57BL/6J (The Jackson Laboratory)	male	10–20	*Gpr30*$^{+/+}$, *Gpr30*$^{+/Venus}$	21
		male	10–20	*Gpr30*$^{-/-}$, *Gpr30*$^{-/Venus}$, *Gpr30*$^{Venus/Venus}$	18
		male	10–18	sham	13
Supplementary Fig. 9f	C57BL/6J (The Jackson Laboratory)	male	10–20	*Gpr30*$^{+/+}$, *Gpr30*$^{+/Venus}$	23
		male	10–20	*Gpr30*$^{-/-}$, *Gpr30*$^{-/Venus}$, *Gpr30*$^{Venus/Venus}$	20
		male	10–18	sham	17
Supplementary Fig. 10a	C57BL/6J (The	male	7	*Gpr30*$^{+/+}$	1
		male	7	*Gpr30*$^{Venus/Venus}$	1

	Jackson Laboratory)	male	8	*Gpr30*$^{+/+}$	2
		male	8	*Gpr30*$^{-/-}$	2
		male	8	*Gpr30*$^{+/Venus}$	1
		male	8	*Gpr30*$^{Venus/Venus}$	2
		male	9	*Gpr30*$^{+/+}$	1
		male	9	*Gpr30*$^{-/-}$	2
		male	11	*Gpr30*$^{+/Venus}$	2
		male	11	*Gpr30*$^{Venus/Venus}$	1
Supplementary Fig. 10b	C57BL/6J (The Jackson Laboratory)	male	14	*Gpr30*$^{+/+}$	5
		male	14	*Gpr30*$^{-/-}$	3
		male	14	*Gpr30*$^{+/Venus}$	4
		male	14	*Gpr30*$^{Venus/Venus}$	4
		male	15	*Gpr30*$^{+/+}$	1
		male	15	*Gpr30*$^{+/Venus}$	4
		male	15	*Gpr30*$^{Venus/Venus}$	2
		male	16	*Gpr30*$^{+/Venus}$	3
		male	16	*Gpr30*$^{Venus/Venus}$	2
Supplementary Fig. 10c	C57BL/6J (The Jackson Laboratory)	male	14	*Gpr30*$^{+/+}$	1
		male	14	*Gpr30*$^{-/-}$	1
		male	15	*Gpr30*$^{+/+}$	1
		male	15	*Gpr30*$^{-/-}$	1
		male	18	*Gpr30*$^{+/+}$	2
		male	18	*Gpr30*$^{-/-}$	3
		male	20	*Gpr30*$^{+/+}$	1
		male	20	*Gpr30*$^{-/-}$	1

Source Data of Supplementary Figures

Uncropped blots of Supplementary Fig. 3a

HA

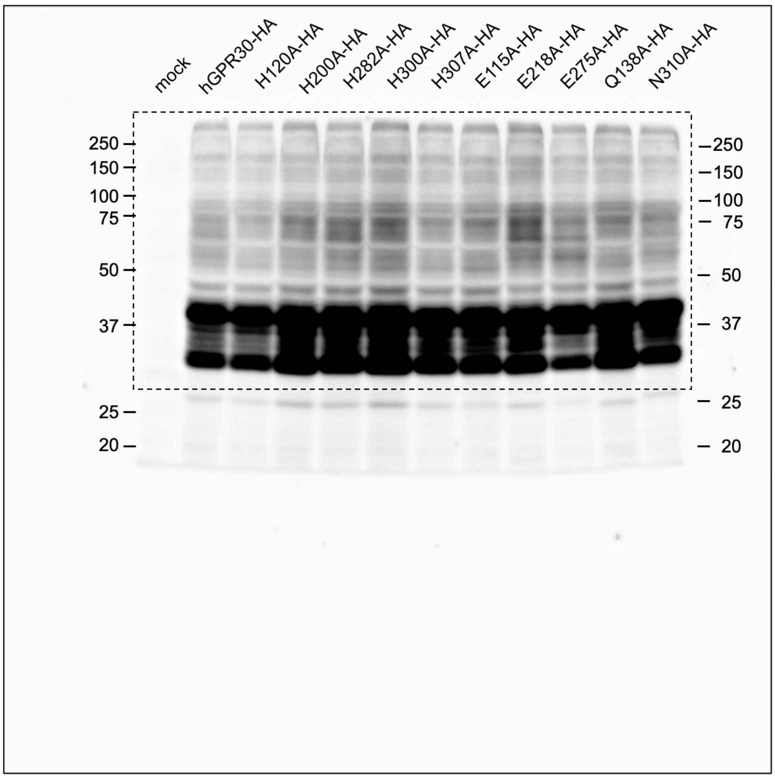

β-actin

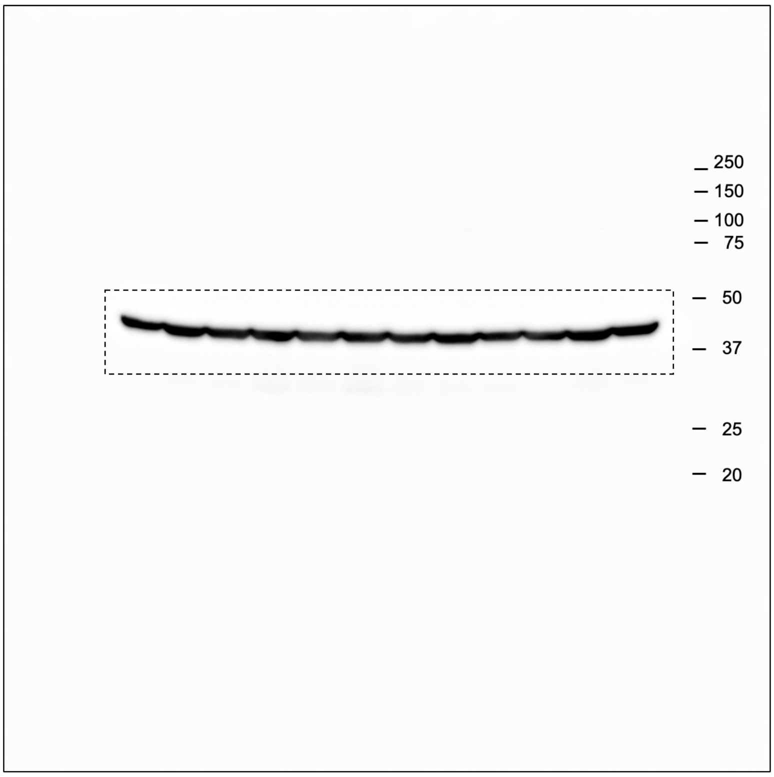

Uncropped blots of Supplementary Fig. 3b

HA

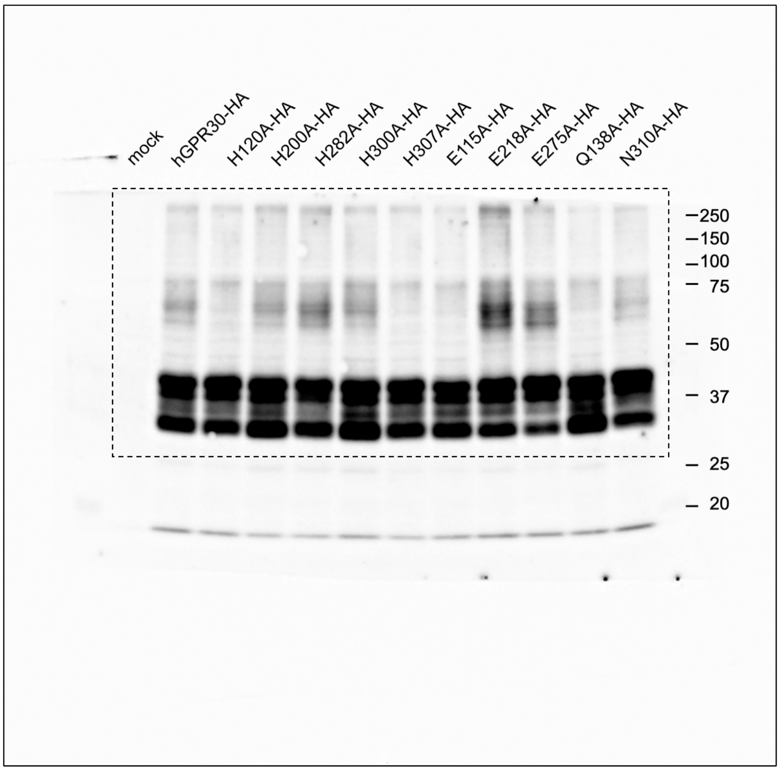

Na-K-ATPase

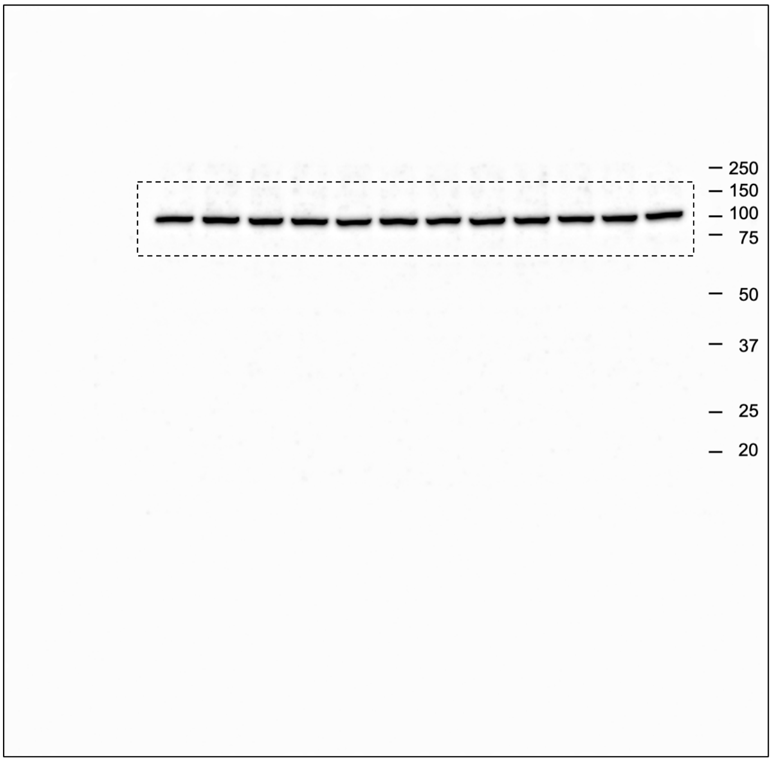

Uncropped blots of Supplementary Fig. 3c

HA

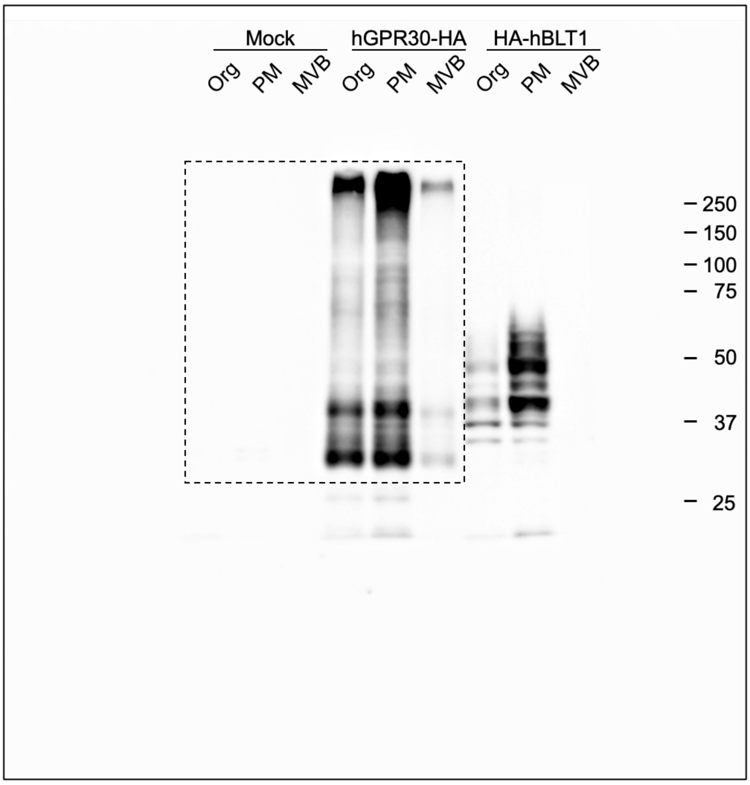

Na-K-ATPase

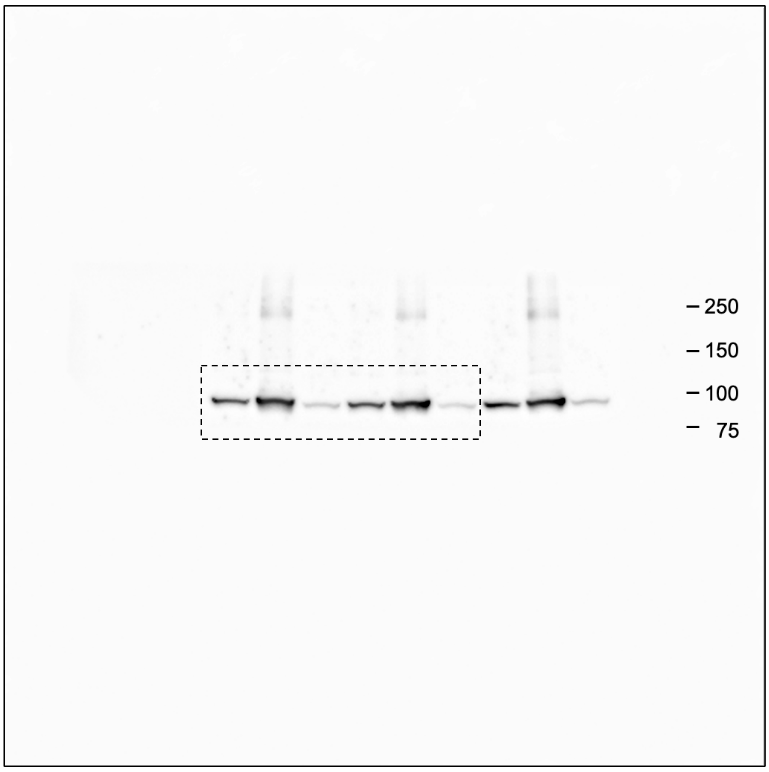

Uncropped blots of Supplementary Fig. 7a

Neuron-specific βIII tubulin

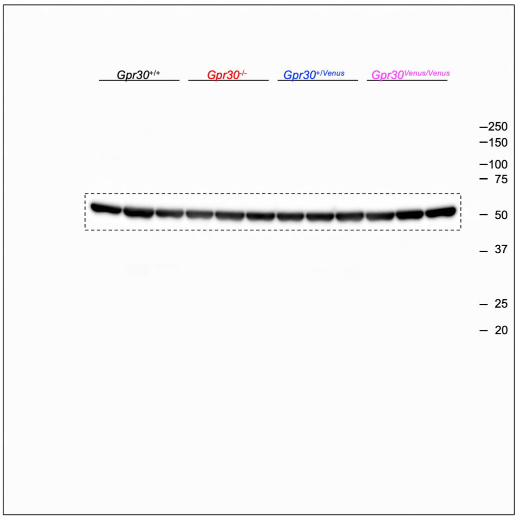

reprobe: β-actin

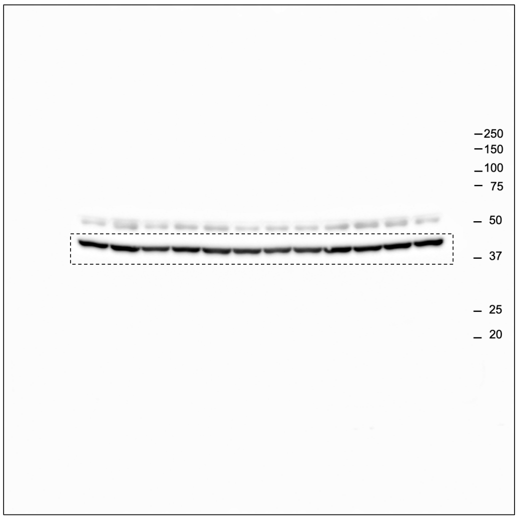

GAD-67

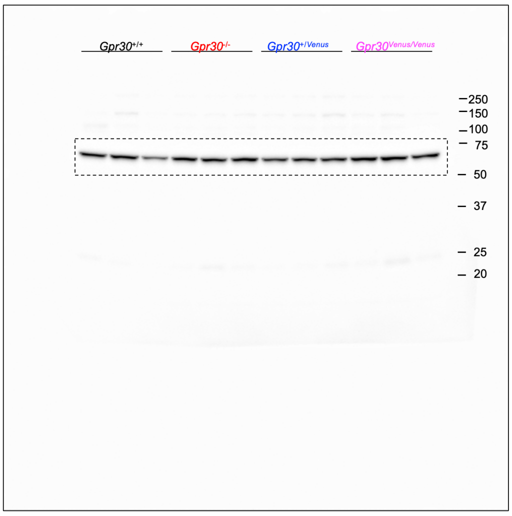

reprobe: β-actin

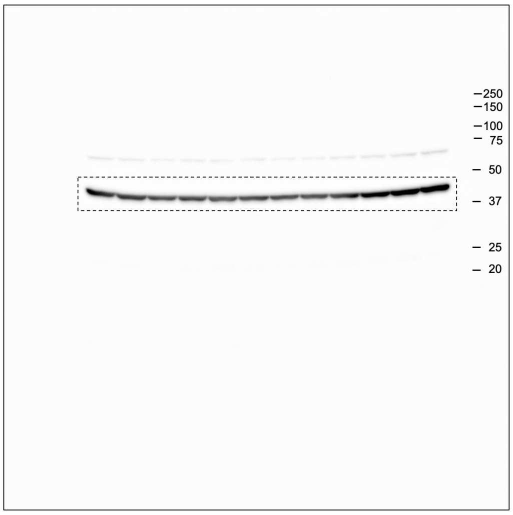

Synaptophysin

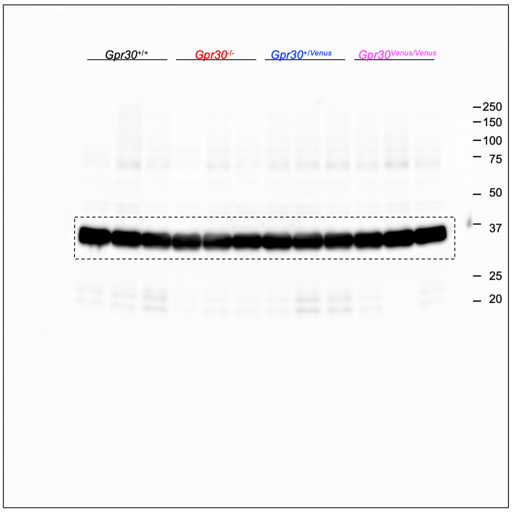

reprobe: β-actin

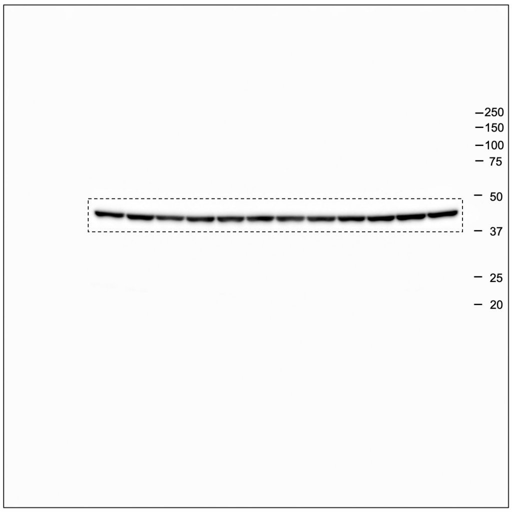

PSD-95

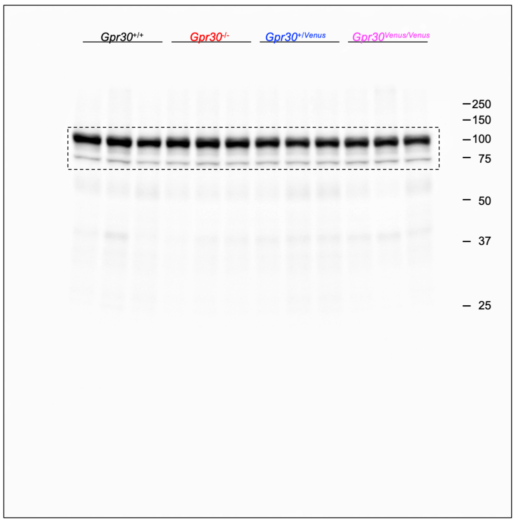

reprobe: β-actin

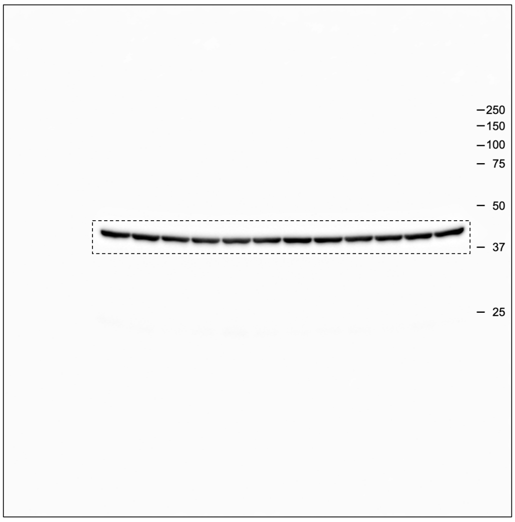

GFAP

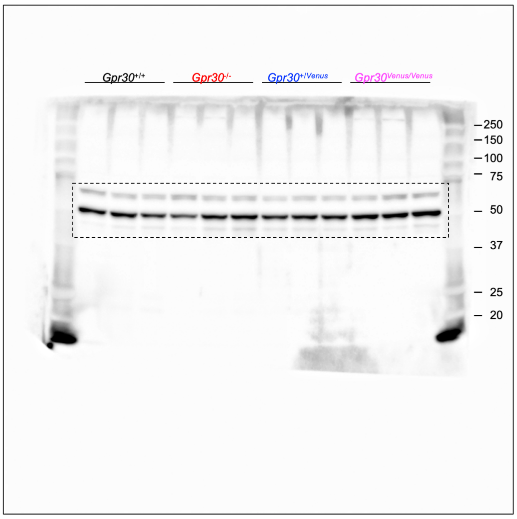

reprobe: β-actin

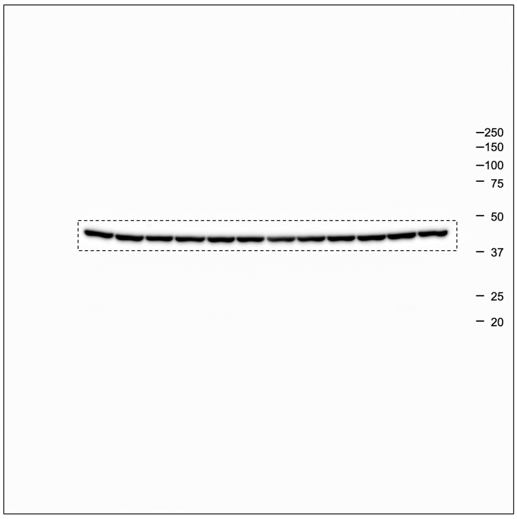

Uncropped blots of Supplementary Fig. 7b

Neuron-specific βIII tubulin

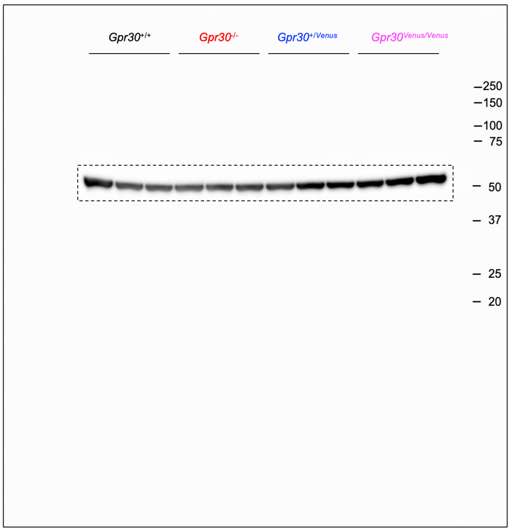

reprobe: β-actin

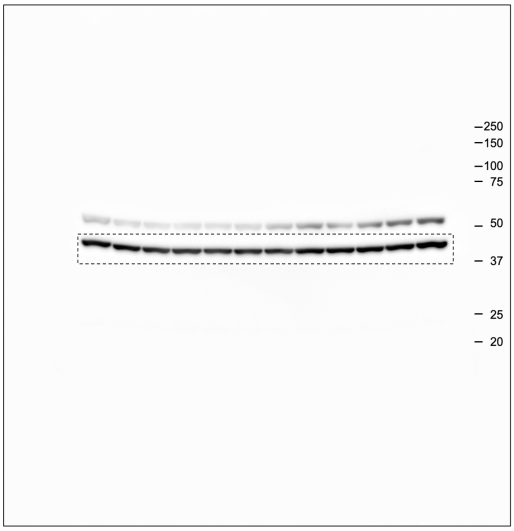

GAD-67

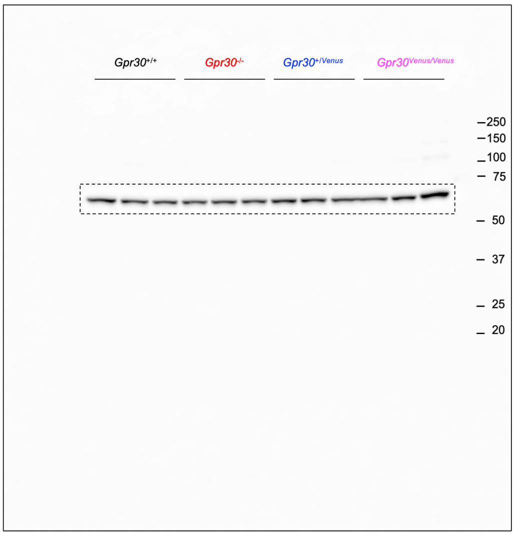

reprobe: β-actin

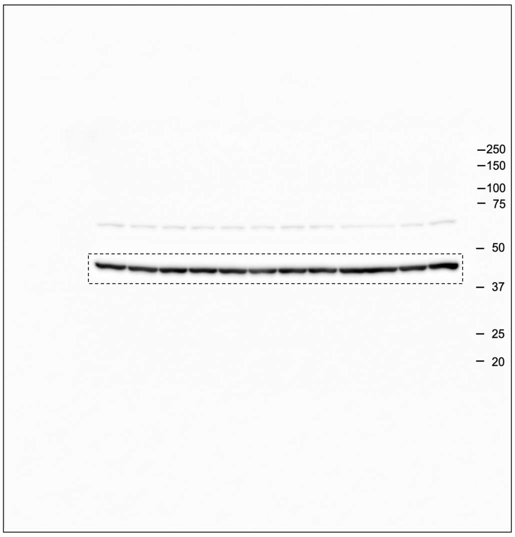

Synaptophysin

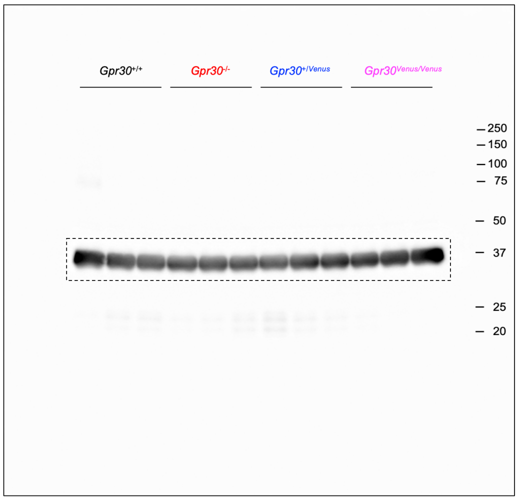

reprobe: β-actin

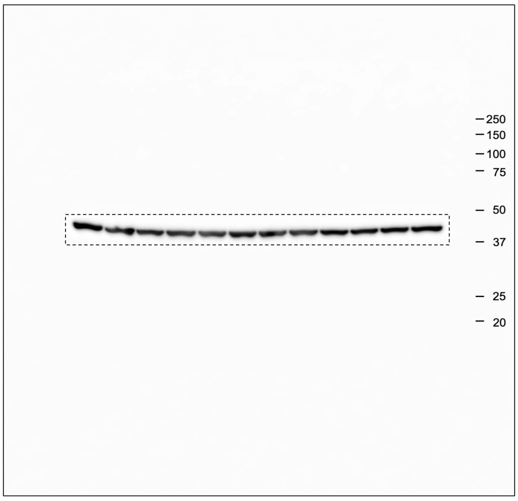

PSD-95

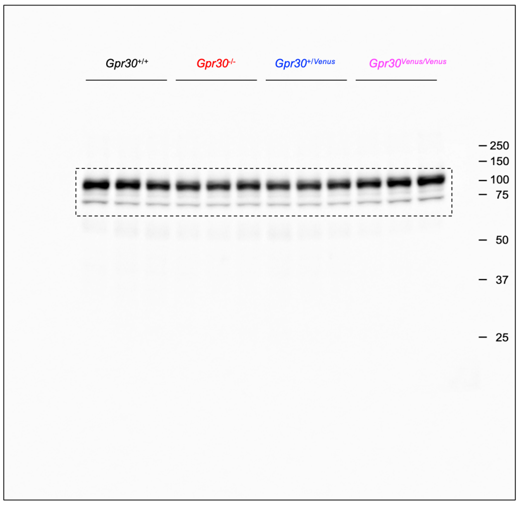

reprobe: β-actin

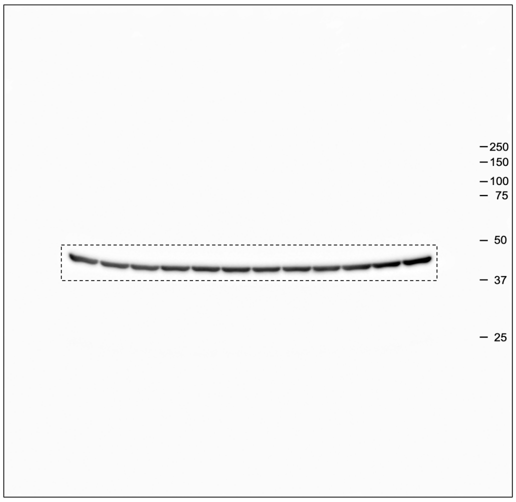

GFAP

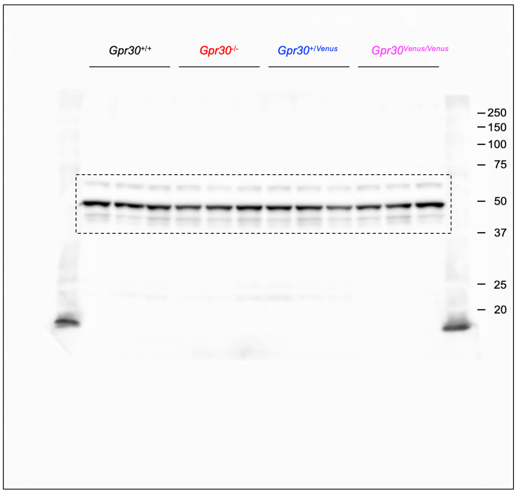

reprobe: β-actin

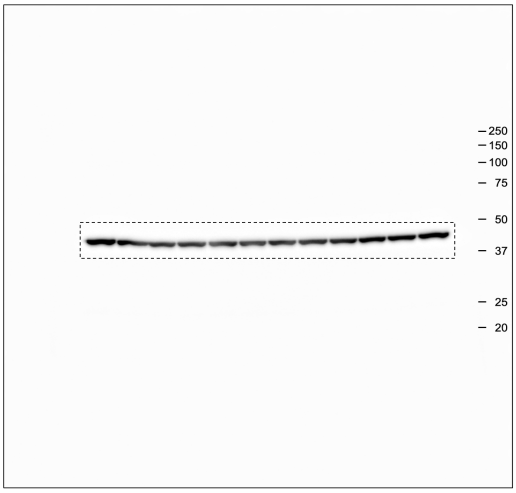

Supplement: Supplementary file 1 — Supplementary Information [file 41467_2024_45579_MOESM1_ESM.pdf]
