# Supplementary material for: Bicarbonate signalling via G protein-coupled receptor regulates ischaemia-reperfusion injury
Source: Nat Commun. 2024 Feb 27;15:1530. doi: 10.1038/s41467-024-45579-3 (PMC10899177; doi:10.1038/s41467-024-45579-3)
Supplement: Supplementary file 3 — Reporting Summary [file 41467_2024_45579_MOESM3_ESM.pdf]

nature portfolio

Corresponding author(s): Airi Jo-Watanabe, Takehiko Yokomizo

Last updated by author(s): Dec 4, 2023

Reporting Summary

Nature Portfolio wishes to improve the reproducibility of the work that we publish. This form provides structure for consistency and transparency in reporting. For further information on Nature Portfolio policies, see our Editorial Policies and the Editorial Policy Checklist.

Statistics

For all statistical analyses, confirm that the following items are present in the figure legend, table legend, main text, or Methods section.

n/a	Confirmed	
☐	☒	The exact sample size (*n*) for each experimental group/condition, given as a discrete number and unit of measurement
☐	☒	A statement on whether measurements were taken from distinct samples or whether the same sample was measured repeatedly
☐	☒	The statistical test(s) used AND whether they are one- or two-sided<br>*Only common tests should be described solely by name; describe more complex techniques in the Methods section.*
☒	☐	A description of all covariates tested
☐	☒	A description of any assumptions or corrections, such as tests of normality and adjustment for multiple comparisons
☐	☒	A full description of the statistical parameters including central tendency (e.g. means) or other basic estimates (e.g. regression coefficient) AND variation (e.g. standard deviation) or associated estimates of uncertainty (e.g. confidence intervals)
☐	☒	For null hypothesis testing, the test statistic (e.g. *F*, *t*, *r*) with confidence intervals, effect sizes, degrees of freedom and *P* value noted<br>*Give P values as exact values whenever suitable.*
☒	☐	For Bayesian analysis, information on the choice of priors and Markov chain Monte Carlo settings
☒	☐	For hierarchical and complex designs, identification of the appropriate level for tests and full reporting of outcomes
☒	☐	Estimates of effect sizes (e.g. Cohen's *d*, Pearson's *r*), indicating how they were calculated

Our web collection on statistics for biologists contains articles on many of the points above.

Software and code

Policy information about availability of computer code

Data collection	Molecular Devices, FlexStation 3; Leica, TCS-SP5; PerkinElmer, Tri-Carb 5110TR; PerkinElmer, MicroBeta TriLux; HAMAMATSU, AQUA COSMOS 2.6.4.0; Cytiva, ImageQuant LAS 4000mini; Vilber, Fusion FX7; Zeiss, ZEN 2 (black edition); Hitachi, HT7700; KEYENCE, BZ-X800 Viewer; OMEGAEAVE, OMEGAFLO, FLO-C1; QuikChange® Primer Design Program by Agilent (https://www.agilent.com/store/primerDesignProgram.jsp); online RNAi design tool (http://www.oligoengine.com);<br>Accession codes, unique identifiers, and web links for publicly available datasets are included in the manuscript.
Data analysis	GraphPad Software, Prism 9.4.0; National Institutes of Health (NIH), ImageJ, version 2.1.0; Zeiss, ZEN 2 (black edition); KEYENCE, BZ-X800 Analyzer, version 1.1.2.4; MathWorks, MATLAB, version R2017b.

For manuscripts utilizing custom algorithms or software that are central to the research but not yet described in published literature, software must be made available to editors and reviewers. We strongly encourage code deposition in a community repository (e.g. GitHub). See the Nature Portfolio guidelines for submitting code & software for further information.

Data

Policy information about availability of data

All manuscripts must include a data availability statement. This statement should provide the following information, where applicable:

- Accession codes, unique identifiers, or web links for publicly available datasets
- A description of any restrictions on data availability
- For clinical datasets or third party data, please ensure that the statement adheres to our policy

Source data files are provided with this paper. Accession codes, unique identifiers, and web links for publicly available datasets are included in the manuscript. The datasets generated during and/or analysed during the current study are as follows and linked to the manuscript; the Psychoactive Drug Screening Program (PDSP) database (https://pdsp.unc.edu/databases/ShaunCell/documents/RegardSupplementalGPCRExpressionRawData.xls), BioGPS (http://biogps.org/), GPCRdb (https://gpcrdb.org), and RNA-sequencing database of mouse brain vascular and perivascular cells (http://betsholtzlab.org/VascularSingleCells/database.html).

Research involving human participants, their data, or biological material

Policy information about studies with human participants or human data. See also policy information about sex, gender (identity/presentation), and sexual orientation and race, ethnicity and racism.

Reporting on sex and gender	N/A
Reporting on race, ethnicity, or other socially relevant groupings	N/A
Population characteristics	N/A
Recruitment	N/A
Ethics oversight	N/A

Note that full information on the approval of the study protocol must also be provided in the manuscript.

Field-specific reporting

Please select the one below that is the best fit for your research. If you are not sure, read the appropriate sections before making your selection.

☒ Life sciences ☐ Behavioural & social sciences ☐ Ecological, evolutionary & environmental sciences

For a reference copy of the document with all sections, see nature.com/documents/nr-reporting-summary-flat.pdf

Life sciences study design

All studies must disclose on these points even when the disclosure is negative.

Sample size	No sample-size calculation was performed.
Data exclusions	Mice died due to MCAO operation were excluded from the analyses.
Replication	Biological and technical replicates in every experiment are shown in Supplementary Table 5 and 6.
Randomization	No methods of randomization were applied.
Blinding	Blinding was applied during the MCAO procedure, mNSS scoring (Fig. 7b, Supplementary Fig. 8a), and MRA analysis (Fig. 8d, e).

Reporting for specific materials, systems and methods

We require information from authors about some types of materials, experimental systems and methods used in many studies. Here, indicate whether each material, system or method listed is relevant to your study. If you are not sure if a list item applies to your research, read the appropriate section before selecting a response.

Materials & experimental systems

n/a	Involved in the study
☐	☒ Antibodies
☐	☒ Eukaryotic cell lines
☒	☐ Palaeontology and archaeology
☐	☒ Animals and other organisms
☒	☐ Clinical data
☒	☐ Dual use research of concern
☒	☐ Plants

Methods

n/a	Involved in the study
☒	☐ ChIP-seq
☒	☐ Flow cytometry
☒	☐ MRI-based neuroimaging

Antibodies

Antibodies used

Cell Signaling Technology, #9101, phospho-p44/42 MAPK (Erk1/2); Santa Cruz Biotechnology, sc-154, ERK2 (C-14); Roche, 11867423001, anti-HA High Affinity (3F10); Cell Signaling Technology, #3010, Na-K-ATPase; Santa Cruz Biotechnology, sc-69879, β-Actin (AC-15); Abcam, ab238135, PSD95 (EPR23124-118);Abcam, ab32127, Synaptophysin (YE269); Abcam, ab26116, GAD67 (K-87); R&D systems, MAB1195, Neuron-specific beta -III Tubulin (TuJ-1); Proteintech, 16825-I-AP, GFAP; Cell Signaling Technology, #7074, anti-rabbit IgG, HRP-linked Antibody; Cell Signaling Technology, #7076, anti-mouse IgG, HRP-linked Antibody; Cytiva, NA935, anti-Rat IgG, HRP-Linked Whole Ab Goat; BD Biosciences, #56435, anti-CD13-Alexa 647; BioLegend, #102515, anti-CD31-Alexa 647; Bio-Rad, #2150-1470, anti-mouse collagen IV; Invitrogen, A10040, Alexa 546-labelled donkey anti-rabbit IgG.

Validation

Validation sheets for all antibodies used in the manuscript are available on the the manufacturer's website and listed below. This information is also shown in the Supplementary Table 4.
Cell Signaling Technology, #9101, phospho-p44/42 MAPK (Erk1/2); https://www.cellsignal.com/products/primary-antibodies/phospho-p44-42-mapk-erk1-2-thr202-tyr204-antibody/9101?country=JP&language=en
Santa Cruz Biotechnology, sc-154, ERK2 (C-14); https://www.scbt.com/p/erk-2-antibody-c-14
Roche, 11867423001, anti-HA High Affinity (3F10); https://www.sigmaaldrich.com/JP/en/product/roche/roahaha
Cell Signaling Technology, #3010, Na-K-ATPase; https://www.cellsignal.com/products/primary-antibodies/na-k-atpase-antibody/3010?country=JP&language=en
Santa Cruz Biotechnology, sc-69879, β-Actin (AC-15); https://www.scbt.com/p/beta-actin-antibody-ac-15?requestFrom=search&bvstate=pg:2/ct:r
Abcam, ab238135, PSD95 (EPR23124-118);https://www.abcam.com/en-mx/products/primary-antibodies/psd95-antibody-epr23124-118-synaptic-marker-ab238135
Abcam, ab32127, Synaptophysin (YE269); https://www.abcam.com/en-th/products/primary-antibodies/synaptophysin-antibody-ye269-ab32127
Abcam, ab26116, GAD67 (K-87); https://www.abcam.com/en-fi/products/primary-antibodies/gad1-gad67-antibody-k-87-ab26116
R&D systems, MAB1195, Neuron-specific beta -III Tubulin (TuJ-1); https://www.rndsystems.com/products/neuron-specific-beta-iii-tubulin-antibody-tuj-1_mab1195
Proteintech, 16825-I-AP, GFAP; https://www.ptglab.com/products/GFAP-Antibody-16825-1-AP.htm
Bio-Rad, #2150-1470, anti-mouse collagen IV; https://www.bio-rad-antibodies.com/polyclonal/mouse-collagen-iv-antibody-2150-1470.html?f=purified
BD Biosciences, #564352, anti-CD13-Alexa 647; https://www.bdbiosciences.com/en-eu/products/reagents/flow-cytometry-reagents/research-reagents/single-color-antibodies-ruo/alexa-fluor-647-rat-anti-mouse-cd13.564352
BioLegend, #102515, anti-CD31-Alexa 647; https://www.biolegend.com/en-us/products/alexa-fluor-647-anti-mouse-cd31-antibody-3094?GroupID=BLG10531
Cell Signaling Technology, #7074, anti-rabbit IgG, HRP-linked Antibody; https://www.cellsignal.com/products/secondary-antibodies/anti-rabbit-igg-hrp-linked-antibody/7074
Cell Signaling Technology, #7076, anti-mouse IgG, HRP-linked Antibody; https://www.cellsignal.com/products/secondary-antibodies/anti-mouse-igg-hrp-linked-antibody/7076
Cytiva, NA935, anti-Rat IgG, HRP-Linked Whole Ab Goat; https://www.citeab.com/antibodies/3288278-na935-amersham-ecl-rat-igg-hrp-linked-whole-antibod
Invitrogen, A10040, Alexa 546-labelled donkey anti-rabbit IgG; https://www.citeab.com/antibodies/2401390-a10040-donkey-anti-rabbit-igg-h-l-highly-cross-ads.

Eukaryotic cell lines

Policy information about cell lines and Sex and Gender in Research

Cell line source(s)

COS-7 (ATCC, CRL1651); HEK293A (female origin; Thermo Fisher Scientific); MCF-7 (JCRB cell bank, JCRB0134); C2C12 (RIKEN BRC, RCB0987); HepG2 (cDNA, a gift from Dr. Tanaka, The University of Tokyo).

Authentication

Authentication of COS-7 is provided by morphology and expression of T antigen; HEK293A by morphology; MCF-7 by morphology and dome-forming character; and C2C12 by morphology, animal PCR, and Simple Sequence Length Polymorphism (mouse).

Mycoplasma contamination

Mycoplasma contamination was not detected in COS-7 (ATCC, CRL1651), MCF-7 (JCRB cell bank, JCRB0134), or C2C12 (RIKEN BRC, RCB0987) at the time of shipment. HEK293A cell lines were not tested for mycoplasma contamination. Mycoplasma contamination is not routinely checked in the laboratory.

Commonly misidentified lines (See ICLAC register)	No commonly misidentified cell lines were used in the study.

Animals and other research organisms

Policy information about studies involving animals; ARRIVE guidelines recommended for reporting animal research, and Sex and Gender in Research

Laboratory animals	C57BL/6J (the Jackson Laboratory) background, three- to four-month-old male mice were used. Mice are housed with 12 h–12 h light–dark cycles, an ambient temperature of 23 ± 2°C , and a humidity of 50 ± 10%, air change rate of 15/h.
Wild animals	Our study did not involve wild animals.
Reporting on sex	We only subjected male mice to the MCAO model in this study, because previous studies suggested that premenopausal females are protected against capillary dysfunction and brain injury in particular conditions.
Field-collected samples	Our study did not involve samples collected from the field.
Ethics oversight	Safety Committee for Recombinant DNA Experiments, Radiation Safety Committee, Institutional Animal Care and Use Committee at Juntendo University School of Medicine (registration number: 1229, approved number: 270213, 280170, 290173, 300154, 310085, 2020148, 2021201, 2022201, and 2023210).

Note that full information on the approval of the study protocol must also be provided in the manuscript.
